# Rootstock-Mediated Genetic Variance in Cadmium Uptake by Juvenile Cacao (*Theobroma cacao* L.) Genotypes, and Its Effect on Growth and Physiology

**DOI:** 10.3389/fpls.2021.777842

**Published:** 2021-12-23

**Authors:** Jessica Fernández-Paz, Andrés J. Cortés, Camila A. Hernández-Varela, Maria Sara Mejía-de-Tafur, Caren Rodriguez-Medina, Virupax C. Baligar

**Affiliations:** ^1^Corporación Colombiana de Investigación Agropecuaria (AGROSAVIA) – C.I Palmira, Palmira, Colombia; ^2^Facultad de Ciencias Agropecuarias, Universidad Nacional de Colombia Sede Palmira, Palmira, Colombia; ^3^Corporación Colombiana de Investigación Agropecuaria (AGROSAVIA) – C.I La Selva, Rionegro, Colombia; ^4^Facultad de Ciencias Agrarias – Departamento de Ciencias Forestales, Universidad Nacional de Colombia Sede Medellín, Medellín, Colombia; ^5^United States Department of Agriculture-Agricultural Research Service-Beltsville Agricultural Research Center, Beltsville, MD, United States

**Keywords:** grafting combinations, cadmium accumulation, cadmium toxicity, rootstock mediated heritability, *Theobroma cacao*

## Abstract

Grafting typically offers a shortcut to breed tree orchards throughout a multidimensional space of traits. Despite an overwhelming spectrum of rootstock-mediated effects on scion traits observed across several species, the exact nature and mechanisms underlying the rootstock-mediated effects on scion traits in cacao (*Theobroma cacao* L.) plants often remain overlooked. Therefore, we aimed to explicitly quantify rootstock-mediated genetic contributions in recombinant juvenile cacao plants across target traits, specifically cadmium (Cd) uptake, and its correlation with growth and physiological traits. Content of chloroplast pigments, fluorescence of chlorophyll *a*, leaf gas exchange, nutrient uptake, and plant biomass were examined across ungrafted saplings and target rootstock × scion combinations in soils with contrasting levels of Cd. This panel considered a total of 320 progenies from open-pollinated half-sib families and reciprocal full-sib progenies (derived from controlled crosses between the reference genotypes IMC67 and PA121). Both family types were used as rootstocks in grafts with two commercial clones (ICS95 and CCN51) commonly grown in Colombia. A pedigree-based best linear unbiased prediction (*A*-BLUP) mixed model was implemented to quantify rootstock-mediated narrow-sense heritability (*h*^2^) for target traits. A Cd effect measured on rootstocks before grafting was observed in plant biomass, nutrient uptake, and content of chloroplast pigments. After grafting, damage to the Photosystem II (PSII) was also evident in some rootstock × scion combinations. Differences in the specific combining ability for Cd uptake were mostly detected in ungrafted rootstocks, or 2 months after grafting with the clonal CCN51 scion. Moderate rootstock effects (*h*^2^> 0.1) were detected before grafting for five growth traits, four nutrient uptake properties, and chlorophylls and carotenoids content (*h*^2^ = 0.19, 95% CI 0.05–0.61, *r* = 0.7). Such rootstock effects faded (*h*^2^< 0.1) when rootstock genotypes were examined in soils without Cd, or 4 months after grafting. These results suggest a pervasive genetic conflict between the rootstock and the scion genotypes, involving the triple rootstock × scion × soil interaction when it refers to Cd and nutrient uptake, early growth, and photosynthetic process in juvenile cacao plants. Overall, deepening on these findings will harness early breeding schemes of cacao rootstock genotypes compatible with commercial clonal scions and adapted to soils enriched with toxic levels of Cd.

## Introduction

Grafting is an ancient technique used to propagate plants vegetatively by combining desirable agronomic traits of the rootstock with those of the scion. It has been used in several plant species targeting a wide spectrum of traits, for instance, resistance to pathogens (Boughalleb et al., 2007; Rivard and Louws, 2008; Spanò et al., 2020); tolerance to abiotic stress factors, such as water deficit, (Liu et al., 2016), heavy metals (Rouphael et al., 2008), and salinity (Yanyan et al., 2018; Suárez-Hernández et al., 2019); improved fruit quality (Davis and Perkins-Veazie, 2005); higher yields (Cardinal et al., 2007); and architectural changes in scions (Eltayb et al., 2014). However, breeding rootstocks for tree crops is slower than scion breeding for the same species. This is due to the long generation times and strong testing requirements of rootstocks, which reduce the opportunity for comprehensively testing their performance against multiple scions and environments.

Conventional propagation by rootstocks and grafting has routinely been used to expand cacao (*Theobroma cacao* L.) cultivation in tropical areas (Ríos, 1957). Seedling rootstocks resistant to *Ceratocystis* spp. and tolerant to acidic soils are typically obtained by open pollination (OP) of the IMC67, PA121, and PA46 reference genotypes in seedling orchards, and are grafted with susceptible productive clones (Palencia Calderon et al., 2007). Yet, only a few studies have evaluated the effect of the rootstock genotype on key agronomic traits such as yield and disease resistance (Yin, 2004; Ribeiro et al., 2016; Asman et al., 2021). For instance, Yin (2004) detected a significant effect of the rootstock on the vigor of cacao scions, but was unable to capture an influence on yield components such as bean weight and number of beans per pod. In line with these results, Asman et al. (2021) observed little rootstock effects on the scion’s resistance to vascular streak dieback, caused by *Ceratobasidium theobromae*. On the other hand, Ribeiro et al. (2016) observed a significant influence of the rootstock × scion interaction on the scion’s resistance to witches’s broom disease caused by *Moniliophthora perniciosa*, which allowed the identification of an elite rootstock genotype with a positive effect on scion disease resistance trait.

Despite the long time during which grafting has been used in the vegetative propagation of *T. cacao*, the rootstock effects on the expression of key scion’s agronomic traits remains poorly understood, among these, cadmium (Cd) accumulation. Content of Cd in cacao products is one of the most limiting factors for cocoa sale in international markets. Cadmium, a heavy metal that causes health problems, accumulates in the seeds of *T. cacao*, which are the raw material to produce chocolate (Barraza et al., 2017). Considering that Cd can accumulate in the human body, starting on January 1, 2019 the European Union (EU) began to control the maximum Cd concentrations allowed in chocolate and cocoa products imported to the EU (European Commission, 2014). Other countries are expected to implement similar regulations to Cd concentration in cocoa products (Vanderschueren et al., 2021), which has generated concern in countries where levels of bioavailable Cd for the plant have been detected in soil. Particularly, high Cd concentrations in cacao beans have been reported in plantations from South America (Chavez et al., 2015; Arévalo-Gardini et al., 2017; Vanderschueren et al., 2019), which has been correlated with the naturally high Cd content in the young soils of this region (Argüello et al., 2019). The Cd concentration in cacao bean is related to soil properties such as total soil Cd, pH, percentage of organic carbon, and oxalate extractable manganese (Argüello et al., 2019). Cd adversely affects growth, photosynthetic process, nutrient uptake, content of chloroplast pigments, cell structure, antioxidative metabolism, and gene expression (Dias et al., 2012; Jiao et al., 2012; Borek et al., 2013; Saidi et al., 2013; Castro et al., 2015; Farooq et al., 2016; Pereira de Araújo et al., 2017).

Cadmium enters root cells through ion channels for Zn, Fe, Mn, and Ca (Song et al., 2017). Transporter gene families like zinc-iron permease (ZIP), natural resistance-associated macrophage proteins (NRAMPs), and heavy metal transporting ATPases (HMAs) have been associated with uptake and translocation of Cd in plants (Vanderschueren et al., 2021). According to some studies, TcNRAMP5 may play a role in the regulation of Cd uptake in cacao plants (Ullah et al., 2018; Moore et al., 2020), whereas HMA-family proteins may contribute to Cd sequestration (Moore et al., 2020). However, the information regarding the specific role of transporter genes in cacao is still limited, as well as the regulatory mechanisms of Cd translocation to the shoot. Within the roots, Cd can be transported in plants through a symplasmic pathway. Cd is later loaded from the symplasm into the xylem (Lux et al., 2011). Movement of Cd from the roots to above-ground tissues depends on the plant mechanisms for vacuolar sequestration, xylem loading, and xylem to phloem transfer (Vanderschueren et al., 2021). In soybean and eggplant, the effect of the root system on the accumulation of Cd in the aerial part of the plant has been demonstrated by grafting cultivars on genotypes that differ in their accumulation of Cd (Sugiyama et al., 2007; Arao et al., 2008). The feasibility of mitigating Cd uptake and accumulation through grafting of highly productive scions on top of rootstocks with low Cd uptake capability has been suggested for *T. cacao* (Lewis et al., 2018; Engbersen et al., 2019). However, as far as we are aware, such effect has not yet been experimentally demonstrated. Further investigation is needed to elucidate the effect of the rootstock in Cd accumulation in cacao. Rootstock genotype may produce unpredicted responses in grafted plants exposed to stressful Cd conditions compared with self-rooted plants (Savvas et al., 2010).

Assessing the effect of rootstock genotypes or specific rootstock × scion combinations on the accumulation of Cd in plant tissues, and the overall plant tolerance to heavy elements, is therefore key for the establishment of new cacao plantations in regions with high levels of available Cd in soil. Therefore, the aim of this study was to quantify the inheritance of the rootstock effects on the scion’s Cd accumulation, as well as associated growth and physiological traits of juvenile cacao plants. Both open-pollinated (OP) and reciprocal full-sib progenies (derived from controlled crosses between the reference genotypes IMC67 and PA121) were used as rootstocks in grafts with two commercial and widely adapted clones (ICS95 and CCN51) in Colombia. Content of chloroplast pigments, fluorescence of chlorophyll *a*, leaf gas exchange, ion leakage, protein content, nutrient uptake, and plant biomass were also examined across rootstock × scion combinations in soils with contrasting levels of Cd. We hypothesize that due to the complex acquisition and transportation of Cd from the root system throughout the plant, there is a potential to harness rootstock-driven genetic variance for Cd uptake as part of early selection schemes at seed orchards, nurseries, and ungrafted saplings.

## Materials and Methods

### Location

The experiment was established under greenhouse conditions at the Palmira Research Station of AGROSAVIA, located in Palmira (3°31′12″N, 76°19′50″W), province of Valle del Cauca, Colombia, at an altitude of 1,001 masl. The climatic conditions during the experiment were recorded using a weather station WatchDog 1000 series Micro Station. Average annual temperature was 26°C, and average relative humidity was 65%.

### Soil Substrate

Soil collected in a cacao growing region of southwest Colombia (pH 4.5) had a natural total Cd content of 0.43 mg kg^–1^. The collected soil was air-dried and passed through a 2-mm mesh sieve. Cd was added to half of the sieved soil using an aqueous solution of Cd(NO_3_)_2_. Cd-spiked soil was incubated at field capacity for 1 month in a greenhouse. During the incubation time, the soil was constantly mixed and maintained at field capacity. The other half of the sieved soil was kept untouched as control treatment.

Greenhouse substrate was prepared by mixing Cd-spiked soil with rice husk and sand in a ratio of 3:1:1 to reach a final content of 7.49 mg Cd kg^–1^, as determined by inductively coupled plasma optical emission spectrometry (Thermo Scientific ICAP 6500). Greenhouse substrate was also prepared using soil without the addition of Cd. According to soil analysis, carried out before the establishment of the experiment, the total nitrogen content in the substrate was 0.4 and 0.5% for soil without addition of Cd and soil enriched with Cd, respectively, which shows that there was no advantage in the concentration of nitrogen in the treatment with addition of Cd.

### Plant Material

Plant material was obtained from the Colombian Cacao Germplasm Bank. Full-sib progenies obtained from the crossing between IMC67 **×** PA121, and its reciprocal cross, were established in Cd-spiked soil, as well as in soil without addition of Cd. Progenies were subsequently evaluated for plant growth, and physiological and nutritional parameters. Meanwhile, a total of two OP half-sib seedling families of IMC67 and PA121 were also considered. These accessions, PA121 and IMC67, corresponded to genotypes recommended as rootstocks in Colombia due to their resistance to pathogens and adaptation to soils with acidic pH (Palencia Calderon et al., 2007). The mucilage of the seed was removed by gently rubbing with sawdust. Seeds were sown in plastic conical tubes containing sand and grown under these conditions for 2 months. After this period, well-developed seedlings were transplanted into black polyethylene bags of 45 cm high and 29 cm wide, containing 10 kg greenhouse substrate with and without the addition of Cd. A commercial fertilizer containing N, P, K, and Mg was applied.

The experiment was established in a randomized complete block design with four replicates arranged in a 4 **×** 2 factorial design and 10 plants per experimental unit. The treatments corresponded to OP families IMC67 and PA121, as well as IMC67 **×** PA121 and PA121 **×** IMC67 progenies from controlled crosses established in soil with and without the addition of Cd. Seedlings were grown on the Cd treatments for 5 months and then evaluated for plant growth, physiological, and nutritional parameters at the end of this period.

Five months after Cd treatment, budwoods of the commercial cultivars ICS95 and CCN51 were grafted onto the seedling rootstocks using the top grafting technique as described by Isele et al. (2020). Taking into consideration that each experimental unit had 10 plants, two of each were evaluated without grafting, four of the remaining plants were grafted with ICS95, and the other four with CCN51. Two and 4 months after grafting, rootstock × scion combinations were evaluated for plant growth, physiological, and nutritional parameters.

### Chlorophyll Fluorescence

Chlorophyll fluorescence was determined by means of a portable optical pulse fluorometer (Opti-Sciences OS30P+). The measurements were made between 7:00 and 11:00 on young, fully expanded, healthy, and photosynthetically active leaves of two plants per experimental unit, usually the third or fourth leaf from the apex of the plants. The leaf was adapted to darkness, using suitable clips, for a period of 30 min and subsequently illuminated with a saturating actinic light pulse of 3,500 μmol m^–2^ s^–1^ for 1 s. The following parameters were evaluated: initial fluorescence (F_0_), maximum fluorescence (F_m_), and the maximum quantum yield of PSII (F_v_/F_m_), obtaining two data per plant.

### Leaf Gas Exchange

Net photosynthetic rate (P_N_), stomatal conductance to water vapor (g_s_), leaf transpiration (E), internal carbon dioxide concentration (Ci), and instantaneous water use efficiency (WUE_INST_) were measured on the same leaf used to determined chlorophyll fluorescence. Gas exchange measurements were performed between 7:00 and 11:00 using an open system portable gas analyzer (ADC model LCpro+), natural light intensity between 250 and 450 μmol m^–2^ s^–1^ and atmospheric CO_2_ of 390 μmol (CO_2_) mol^–1^. The leaf area sampled was 6.25 cm^2^. Two data points per plant were obtained.

### Chloroplast Pigments

Chlorophyll *a*, chlorophyll *b*, their ratio, total chlorophyll, and carotenoids were quantified in the same leaves used to measure gas exchange and fluorescence emission. Pigments were extracted from three disk-shaped leaf segments per plant using cold 80% ammoniacal acetone (4°C), following the protocol described by Melgarejo et al. (2010) and calculated according to Lichtenthaler (1987). The area of each disk was 0.78 cm^2^. The absorbance of the extracts was determined with a spectrophotometer (PerkinElmer Lambda 25) using the following wavelengths: 663, 647, and 470 nm to calculate the concentrations of chlorophyll *a*, chlorophyll *b*, and total carotenoids, respectively (Lichtenthaler, 1987).

Pigment concentration was calculated from the following equations:

Ca=12.25AA663-2.79647

Cb=21.5AA647-5.1663

C(a+b)=7,15AA663+18.7647

Cx+c=1000ACa470-1.82-85,02Cb/198

where Ca stands for chlorophyll *a*, Cb stands for chlorophyll *b*, C (a + b) stands for total chlorophyll, and Cx + c stands for carotenoids.

### Electrolyte Leakage

To determine electrolyte leakage (EL), 0.78 cm^2^ leaf disks were taken from the same leaf used to determine the content of chloroplast pigments. Three disks were obtained from each leaf sample, washed with distilled water, and then arranged in 15 mL conical Falcon tubes containing 3 mL of deionized water. The samples were incubated at room temperature (22°C) on a shaker for a period of 5 h and the electrical conductivity was recorded at the end of this period (EC1). The Falcon tubes were then taken to a water bath at 80°C for 10 min and electrical conductivity (EC2) recorded again after cooling the bathing solution to 22°C. The loss of electrolytes (EL) was calculated using the following formula:

$$EL=\frac{EC1}{EC2}\times 100$$

EC1 corresponds to the electrical conductivity measured after 5 h in deionized water. EC2 corresponds to the electrical conductivity measured after incubation at 80°C.

### Plant Growth and Biomass

Once the physiological parameters were evaluated during morning hours, seedling rootstocks, and also grafting combinations, were harvested for shoot and root lengths measures. Plants were then divided into roots, stem, and leaves. Plant tissue was washed with distilled water and gently dried with absorbent towels. Fresh weight of each plant portion was then determined. To wash the excess of Cd adhered to the roots, these were immersed for 3 min in a solution containing 5 mM EDTA and 20 mM TRIS (pH 8.0) with constant agitation followed by three washes with distilled water for 3 min (Nguyen et al., 2016). The reason why roots were washed was to prevent externally adhered Cd from being quantified as if it were inside the root system. Samples were placed in paper bags and oven-dried at 60°C for 72 h, and after this period dry weight was determined.

### Leaf Area

Leaf area was measured using an electronic area meter (LICOR-3000) immediately after harvesting leaf tissue.

### Protein Content

Approximately 100 mg of leaf tissue were macerated in liquid nitrogen. Phosphate buffer (0.05 M) was then added, and the samples centrifuged for 30 min at 12,000 **×** *g* and 4°C. Total soluble protein content was measured by the Coomassie blue method according to Bradford (1976) using BSA as standard in a microplate spectrophotometer (BioTek EPOCH) at 595 nm.

### Cadmium and Mineral Nutrients Content

Dried plant material was ground in a mill (Thomas Wiley) and submitted to nitric–perchloric digestion assisted by microwave (Milestone UltraWave). Mineral elements such as Cd, Ca, Mg, Fe, Zn, Cu, and Mn were determined by atomic absorption spectrophotometry, P by colorimetry, and K by flame emission photometry (Isaac and Kerber, 1971). Nitrogen content was determined by the Kjeldahl method (Bradstreet, 1954).

### Data Analyses and Rootstock-Mediated Heritability Scores

Trait variation across families was explicitly compared using a mixed linear model in which family identity and Cd treatment were indexed as fixed effect and repetition as random effect. Second, rootstock-mediated additive genetic variance (i.e., narrow sense hereditability) was obtained for each trait using a best linear unbiased prediction (BLUP) mixed model that relied on the pedigree information (i.e., a pedigree relationship matrix for an *A*-BLUP model) from the rootstocks (half-sib families for OP, and full-sib families for controlled crossing schemes, allowing for a 5% of maternal effects). The corresponding linear mixed model, following Arenas et al. (2021), was fitted as in:

y=Xb+Za+e

where *y* is the phenotypic trait vector, *a* is a vector of individual random additive genetic effects with a normal distribution so that *a* ∼ N(0, *A*σ_*a*_^2^) with *A* being the pedigree relationship matrix among rootstocks and *σ_*a*_^2^* the additive genetic variance (Müller et al., 2017), *b* is a vector of fixed effects (i.e., intercept or general mean, and experimental site effects), *e* is the vector of residual error effect, and *X* and *Z* are the corresponding incidence matrices for fixed effects and additive genetic effects (Chen et al., 2018; Gutierrez et al., 2018), respectively.

To aid interpretability while accounting for clonal scion differences and *in situ* environmental (soil) variation (of major importance in Cd fixation), genetic parameters for all phenotypic traits were individually estimated for each environment and scion, rather than including the latter as explicit fixed effects within the model. All models implemented the reproducing kernel Hilbert space (RKHS) method throughout the BGLR (Bayesian Generalized Linear Regression) package (Pérez-Rodríguez and de los Campos, 2014) in R v.3.4.4 (R Core Team). RKHS is a semiparametric approach to infer a given function without making a strong *a priori* assumption about the distribution of effects (Cuevas et al., 2016). RKHS was executed using a Gibbs sampling with 10,000 iterations after discarding the 5,000 initial steps as burn-in. A thinning interval of 10 was implemented for data recording. Convergence of posterior distributions was verified using trace plots, whereas rootstock-mediated specific combining abilities, original trait correlations (Supplementary Figure 1), and *A* input pedigree relationship matrices (Supplementary Figure 2) were respectively checked using the R (R Core Team) functions *lme* (from the *nlme* package, treating families as fixed effects and repetitions as random effects), *cor.test*, and *heatmap*.

Narrow sense rootstock-mediated heritability (*h*^2^) was then computed using the additive (*σ_*a*_^2^*) and residual (*σ_*e*_^2^*) variances, following de los Campos et al. (2015), as in:

$$h^{2}=\sigma_{a}^{2}/\left(\sigma_{a}^{2}+\sigma_{e}^{2}\right)$$

Variance within experimental units was captured by the residual variance term because it did not correspond to an additive variance component. Narrow sense rootstock-mediated heritability (*h*^2^) estimates were summarized using the median and the 95% confidence interval from the BGLR’s posterior distribution. Overall models’ fits were examined by computing the prediction ability (*r*_y_) estimated for each trait as the Pearson’s correlation between the observed and the predicted (i.e., breeding value deviation from the overall mean) trait values (Müller et al., 2017; Zhang et al., 2019).

## Results

### Cadmium Accumulation

**FIGURE 1:**
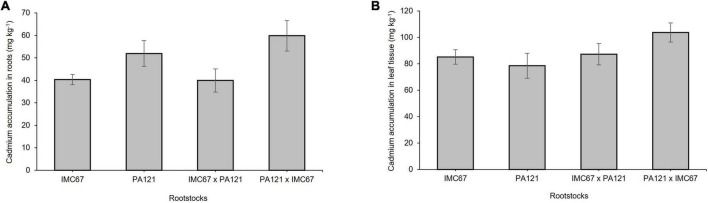
Cadmium accumulation across four ungrafted seedling rootstocks grown in soil enriched with Cd (7.49 mg Cd kg^–1^ soil) 5 months after treatment. **(A)** Cadmium accumulation in roots. **(B)** Cadmium accumulation in leaves. Data are mean values ± SE (*n* = 8).

Significant differences in the accumulation of Cd in roots before grafting were observed between the seedling rootstocks established in Cd-spiked soil. Accumulation of Cd in the roots of PA121 **×** IMC67 full-sib progenies was significantly higher (*p* < 0.05) than in IMC67 OP half-sib and IMC67 **×** PA121 full-sib families (Figure 1A). On the other hand, no significant differences were observed in the accumulation of Cd in leaves between the family rootstocks subjected to stress by Cd (Figure 1B).

**FIGURE 2:**
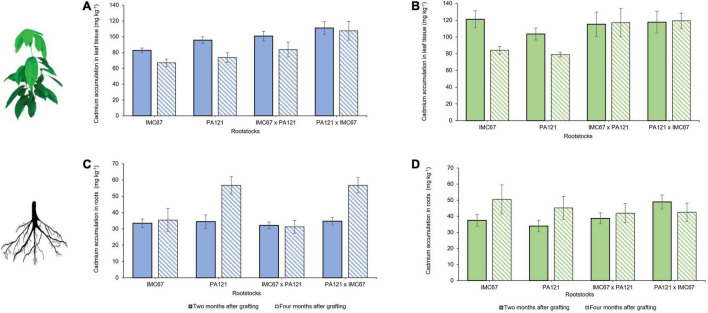
Cadmium accumulation in rootstock × scion combinations 2 and 4 months after grafting. Seedling rootstocks families were established in soil enriched with Cd (7.49 mg Cd kg^–1^ soil). Panels **(A,C)** depict cadmium accumulation in grafting combinations having ICS95 as scion. Panels **(B,D)** show Cd accumulation in grafting combinations having CCN51 as scion. Data are mean values ± SE (*n* = 8).

Cadmium accumulation in both rootstocks and scions was quantified 2 and 4 months after grafting the ICS95 and CCN51 clonal scions on the seedling rootstock families (Figure 2). At 2 months after grafting, lower accumulation of Cd in leaf tissue was observed when the ICS95 clone was grafted on the IMC67 OP half-sib progenies (Figure 2A). However, no significant differences in Cd accumulation were observed between any of the rootstock families on which ICS95 was grafted (Figure 2C). Four months after grafting, a greater accumulation of Cd in leaf tissue of the ICS95 clonal scion grafted on PA121 **×** IMC67 full-sib rootstocks was observed (Figure 2A). For this case, lower accumulation of Cd in roots of the IMC67 OP half-sib and IMC67 **×** PA121 full-sub families was observed when grafted with the ICS95 clonal scion (Figure 2C).

On the other hand, no significant differences were observed in Cd accumulation of the CCN51 clonal scion when grafted on any of the evaluated rootstocks 2 months after grafting (Figure 2B). However, a greater accumulation of Cd was observed in PA121 **×** IMC67 full-sib progenies when they were grafted with the CCN51 scion (Figure 2D). Four months after grafting, a greater accumulation of Cd was observed in CCN51 clonal scion grafted on IMC67 **×** PA121 full-sib rootstocks, and its reciprocal progenies (Figure 2B). For this case, no significant differences in Cd accumulation were observed between rootstock families having CCN51 as scion (Figure 2D).

**FIGURE 3:**
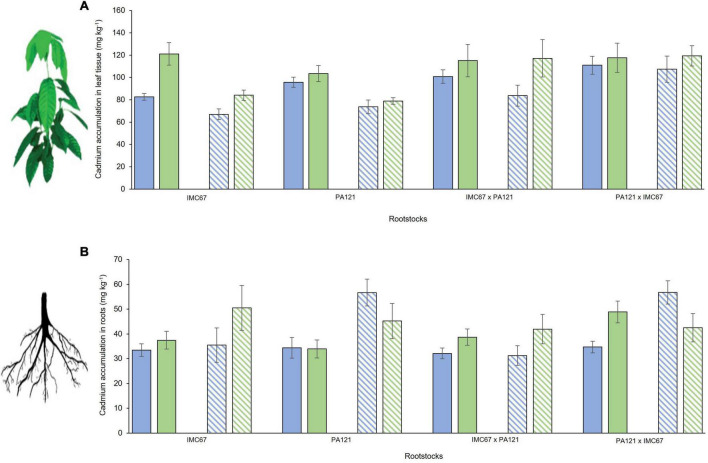
Cadmium accumulation of ICS95 and CCN51 clonal scions grafted on four seedling rootstock families 2 and 4 months after grafting. Seedling rootstocks (*x*-axis) were grown in soil enriched with Cd (7.49 mg Cd kg^–1^ soil). **(A)** Cadmium accumulation in leaf tissue of the ICS95 and CCN51 clonal scions. **(B)** Cadmium accumulation in the roots of the four OP rootstock families when grafted with the ICS95 and CCN51 clones. Blue bars represent grafting combinations with ICS95 as scion 2 months after grafting. Green bars represent grafting combinations with CCN51 as scion 2 months after grafting. Data are mean values ± SE (*n* = 8). Dashed bars show Cd accumulation 4 months after grafting.

Cadmium accumulation in the leaf tissue of the CCN51 scion grafted on IMC67 OP half-sib rootstock progenies, 2 and 4 months after grafting, was significantly higher than for the ICS95 clonal scion (Figure 3A). No significant differences in Cd accumulation were observed between ICS95 and CCN51 when grafted onto any of the other rootstock families at 2 or 4 months after grafting (Figure 3A). Significant differences in Cd accumulation in the roots were observed only for the PA121 × IMC67 full-sib rootstock family. A higher accumulation of Cd was observed when this rootstock family was grafted with the CCN51 scion 2 months after grafting. However, 4 months after grafting a higher accumulation of Cd in the roots was observed when using ICS95 as scion (Figure 3B).

### Plant Growth

**FIGURE 4:**
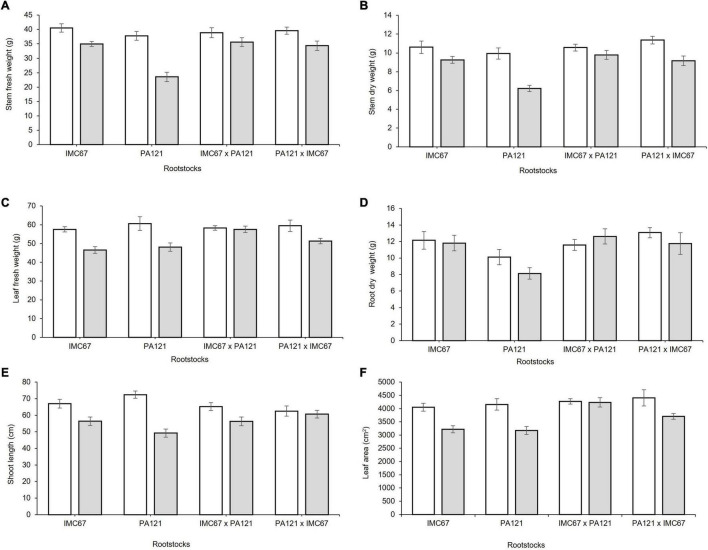
Plant growth of four families of ungrafted seedlings under greenhouse conditions at two different concentrations of Cd in soil, 5 months after cadmium treatment. **(A)** Stem fresh weight, **(B)** stem dry weight, **(C)** leaf fresh weight, **(D)** root dry weight, **(E)** shoot length, and **(F)** leaf area. White bars represent rootstocks grown in soil without addition of Cd (0.43 mg kg^–1^). Gray bars represent rootstocks grown in Cd spiked soil (7.49 mg kg^–1^). Data are mean values ± SE (*n* = 8).

A significant decrease in plant biomass, shoot length, and leaf area was observed 5 months after establishment of the ungrafted seedlings in Cd-spiked soil (Figures 4A–F). Fresh weight of the stem decreased by 13.7, 37.6, 8.3, and 13.2% for the OP half-sib families IMC67 and PA121, and for the full-sib IMC67 **×** PA121 and PA121 **×** IMC67 progenies, respectively, as compared with the control (without Cd addition) (Figure 4A). A greater decrease in stems’ (37.4%) and roots’ (19.5%) dry weight was also observed for PA121 OP half-sib rootstocks subjected to stress by Cd addition in the soil (Figures 4B,D). A similar response was observed for shoot length, for which a decrease of 15.8, 32, 13.6, and 2.8% was observed respectively, for OP half-sib progenies IMC67 and PA121, and full-sib IMC67 **×** PA121 and PA121 **×** IMC67 families (Figure 4E).

**FIGURE 5:**
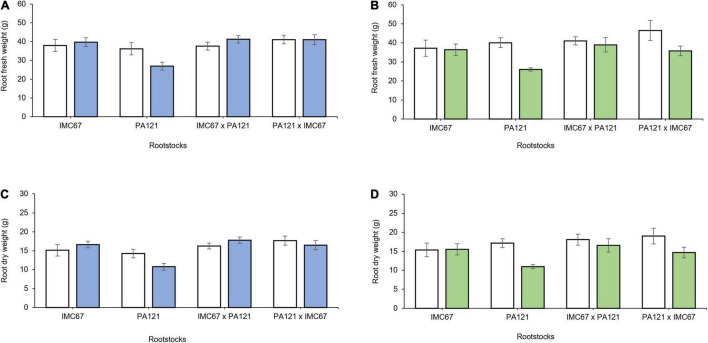
Root biomass of rootstock × scion combinations 2 months after grafting seedling rootstocks subjected to two different concentrations of Cd in soil under greenhouse conditions. Panel **(A,B)** depict fresh root weight of rootstock families having ICS95 and CCN51 as scions. Panel **(C,D)** show dry root weight of OP and full-sib rootstock progenies having ICS95 and CCN51 as scion. White bars represent rootstocks grown in soil without addition of Cd (0.43 mg kg^–1^). Blue bars represent rootstocks grown in Cd spiked soil (7.49 mg kg^–1^) grafted with the ICS95 clonal scion. Green bars represent rootstocks grown in Cd spiked soil (7.49 mg kg^–1^) grafted with the CCN51 clonal scion. Data are mean values ± SE (*n* = 8).

Two months after grafting, significant changes in root biomass were observed for grafting combinations having both ICS95 and CCN51 as scion (Figures 5A–D). Combinations of ICS95 as scion and PA121 half-sib progenies as rootstock showed a significant decrease in root biomass. Specifically fresh and dry root weight fell in 25.5 and 24.6%, respectively, compared with the control (Figures 5A,C). Likewise, 2 months after grafting, PA121 half-sib rootstock families showed a significant decrease in root biomass in combination with the CCN51 clonal scion. For this case, fresh and dry root weight respectively, decreased by 35.13 and 36.15% compared with the control (without Cd addition) (Figures 5B,D).

**FIGURE 6:**
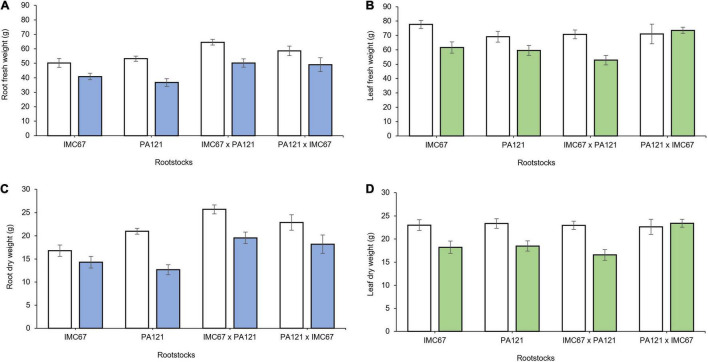
Plant growth of rootstock × scion combinations 4 months after grafting at two different concentrations of Cd in soil under greenhouse conditions. Panels **(A,C)** show fresh and dry root weight of seedling rootstocks grafted with the ICS95 clonal scion. Panels **(B,D)** depict fresh and dry leaf weight of the CCN51 clonal scion across rootstock families. White bars represent rootstocks grown in soil without addition of Cd (0.43 mg kg^−1^). Blue bars represent rootstock families grafted with the ICS95 clonal scion established in Cd spiked soil (7.49 mg kg^–1^). Green bars represent rootstock families grafted with the CCN51 clonal scion established in Cd spiked soil (7.49 mg kg^–1^). Data are mean values ± SE (*n* = 8).

Four months after grafting, a significant decrease in root biomass was also observed in all rootstock families grafted with the ICS95 scion (Figures 6A,C). On the other hand, 4 months after grafting, seedling rootstocks grafted with the CCN51 clonal scion showed significant changes not only in root growth parameters but also in the scion growth. A significant decrease in leaf area and in fresh and dry weight of leaf tissue was observed in the CCN51 scion grafted on all the rootstock families, except when it was grafted on PA121 **×** IMC67 full-sib rootstocks (Figures 6B,D). A decrease in dry root weight of 23.5 and 37.2% was respectively, observed in the OP half-sib rootstock families IMC67 and PA121, both grafted with the CCN51 clonal scion (to be presented in section Rootstock-Mediated Specific Combining Abilities and Heritability Scores in Full-Sib Families).

### Chlorophyll Fluorescence and Leaf Gas Exchange

**FIGURE 7:**
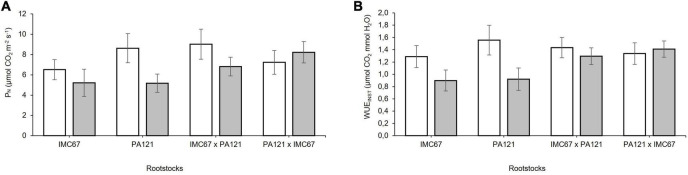
Leaf gas exchange in four seedling rootstock families subjected to two different concentrations of Cd in soil under greenhouse conditions 5 months after cadmium treatment. **(A)** Photosynthetic rate (P_N_), and **(B)** instantaneous water use efficiency (WUE_INST_). White bars represent rootstock families grown in soil without addition of Cd (0.43 mg kg^–1^). Gray bars represent rootstock families grown in Cd spiked soil (7.49 mg kg^–1^). Data are mean values ± SE (*n* = 8).

A decrease in the net photosynthetic rate (*P*_N_) and in instantaneous water use efficiency (WUE_INST_) was observed in response to Cd toxicity before grafting (Figure 7). A significant decrease (*p* < 0.05) in the carbon assimilation rate equivalent to 40.09%, compared with the control, was observed when using PA121 OP half-sib seedling rootstocks (Figure 7A). Water use efficiency respectively, decreased by 30.46 and 40.6% when using IMC67 and PA121 OP half-sib seedling rootstock families, compared with control plants (Figure 7B). There was no effect of Cd on leaf gas exchange variables when using ICS95 and CCN51 as clonal scions, neither at 2 nor at 4 months after grafting.

**FIGURE 8:**
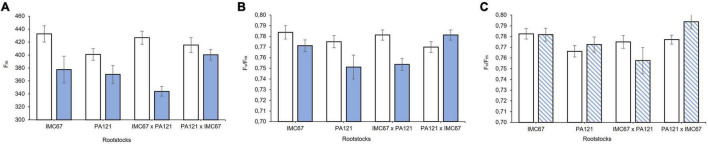
Chlorophyll fluorescence in the ICS95 clonal scion when grafted with 4 different rootstock families at two different concentrations of Cd in soil under greenhouse conditions. **(A)** F_m_ 2 months after grafting. **(B)** F_v_/F_m_ 2 months after grafting. **(C)** F_v_/F_m_ 4 months after grafting. White bars represent rootstock families grown in soil without addition of Cd (0.43 mg kg^–1^). Blue bars represent rootstock families grown in Cd spiked soil (7.49 mg kg^–1^). Dashed bars show Cd accumulation 4 months after grafting. Data are mean values ± SE (*n* = 8).

Variables of chlorophyll *a* fluorescence emission did not show significant differences (*p* < 0.05) between seedling rootstock families established in soil with and without the addition of Cd before grafting (to be presented in section Rootstock-Mediated Specific Combining Abilities and Heritability Scores in Full-Sib Families). However, 2 months after grafting, a significant decrease in F_m_ was observed in ICS95 clonal scion grafted on all the rootstock families. A greater decrease of 19.4% in F_m_ was observed in this clonal scion grafted on IMC67 **×** PA121 full-sib rootstock progenies (Figure 8A). An effect of Cd on F_m_ resulted in a change in F_v_/F_m_ ratio scores. A significant decrease in F_v_/F_m_ was observed in the ICS95 clonal scion grafted on PA121 OP half-sib and IMC67 **×** PA121 full-sib rootstock families 2 months after grafting (Figure 8B). On the other hand, an increase in F_v_/F_m_ ratio was observed in the same clone when grafted on the reciprocal PA121 **×** IMC67 full-sib progenies 2 and 4 months after grafting (Figures 8B,C), which suggests for a maternal effect. There was no significant difference when the clonal scion CCN51 was grafted with the different seedling rootstock families for the variables of chlorophyll fluorescence at 2 or at 4 months after grafting (to be presented in section Rootstock-Mediated Specific Combining Abilities and Heritability Scores in Full-Sib Families).

### Chloroplast Pigments

**FIGURE 9:**
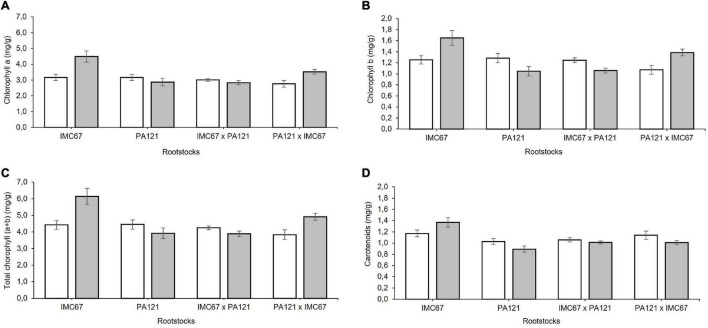
Content of chloroplast pigments of ungrafted seedling families subjected to two different concentrations of Cd in soil under greenhouse conditions 5 months after cadmium addition. **(A)** Content of chlorophyll *a*. **(B)** Content of Chlorophyll *b*. **(C)** Content of total Chlorophyll. **(D)** Content of carotenoids. White bars represent seedling families grown in soil without addition of Cd (0.43 mg kg^–1^). Gray bars represent seedling progenies grown in Cd spiked soil (7.49 mg kg^–1^). Data are mean values ± SE (*n* = 8).

Significant changes in the content of chloroplast pigments were observed in the ungrafted seedlings 5 months after establishment in Cd-spiked soil (Figure 9). A different effect of Cd was observed depending on the rootstock. A significant increase in chlorophyll *a* equivalent to 41.6 and 21.8% was respectively, observed in IMC67 OP half-sib and PA121 **×** IMC67 full-sib progenies, compared with the control (Figure 9A). IMC67 OP half-sib and PA121 **×** IMC67 full-sib families also showed a significant increase in chlorophyll *b* content respectively, equivalent to 32 and 29% (Figure 9B). Changes in chlorophyll *a* and *b* resulted in changes in total chlorophyll, accordingly. A significant increase of total chlorophyll was observed in IMC67 OP half-sib and PA121 **×** IMC67 full-sib families (Figure 9C). The carotenoid content also increased by 17% in IMC67 OP half-sib progenies 5 months after Cd addition. The content of carotenoids in the remaining families decreased (Figure 9D).

**FIGURE 10:**
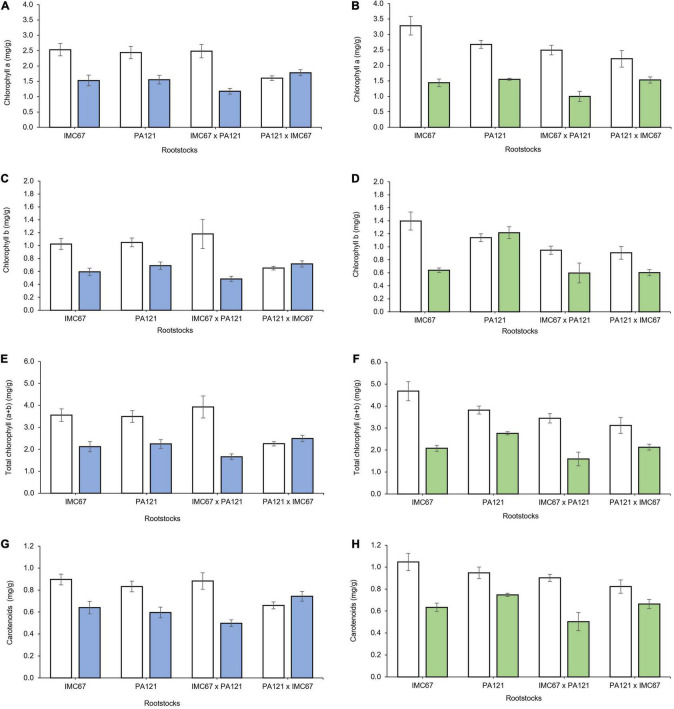
Content of chloroplast pigments in ICS95 and CCN51 clonal scions 2 months after grafting on four different seedling rootstock families subjected to two different concentrations of Cd in soil under greenhouse conditions. Panels **(A,B)** show content of Chlorophyll a of ICS95 and CCN51 clonal scions. Panels **(C,D)** depict content of Chlorophyll b of ICS95 and CCN51 clonal scions. Panels **(E,F)** exhibit content of total Chlorophyll of ICS95 and CCN51. Panels **(G,H)** show content of carotenoids of ICS95 and CCN51 clonal scions. White bars represent rootstocks × scion combinations grown in soil without addition of cadmium (0.43 mg kg^–1^). Blue bars show seedling rootstock families grafted with the ICS95 clonal scion grown in Cd spiked soil (7.49 mg kg^–1^). Green bars depict seedling rootstock families grafted with the CCN51 clonal scion grown in Cd spiked soil (7.49 mg kg^–1^). Data are mean values ± SE (*n* = 8).

Two months after grafting, a significant decrease in all chloroplast pigments was observed across seedling rootstock families grafted with both ICS95 and CCN51 clonal scions (Figure 10).

### Electrolyte Leakage

**FIGURE 11:**
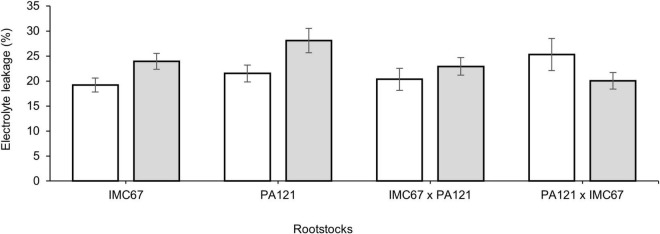
Electrolyte leakage in four ungrafted seedling families subjected to two different concentrations of Cd in soil under greenhouse conditions 5 months after Cd addition. White bars represent ungrafted seedling families grown in soil without the addition of Cd (0.43 mg kg^–1^). Gray bars represent ungrafted seedling families grown in Cd-spiked soil (7.49 mg kg^–1^). Data are mean values ± SE (*n* = 8).

A significant increase of 24.7 and 30.6% in electrolyte leakage was observed in ungrafted IMC67 and PA121 OP half-sib families established in Cd-spiked soil, suggesting cell membrane damage in these rootstocks (Figure 11). No significant difference was observed in electrolyte leakage after grafting with the ICS95 and CCN51 clonal scions in Cd-spiked soil (Supplementary Table 5).

### Protein Content

**FIGURE 12:**
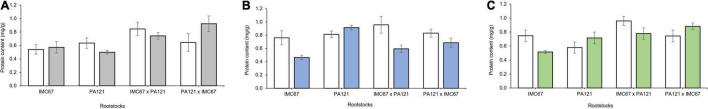
Protein content in leaf tissue of rootstocks and grafting combinations subjected to two different concentrations of Cd in soil under greenhouse conditions. **(A)** Protein content in leaf tissue of ungrafted seedling families 5 months after Cd addition. **(B)** Protein content in leaf tissue of the ICS95 clonal scion 2 months after grafting with four different rootstock families. **(C)** Protein content in leaf tissue of the CCN51 clonal scion 2 months after grafting with four different rootstock families. White bars represent families grown in soil without addition of Cd (0.43 mg kg^–1^). Gray bars represent ungrafted seedling families grown in Cd-spiked soil (7.49 mg kg^–1^). Blue bars represent seedling rootstock families grafted with the ICS95 clonal scions in Cd-spiked soil (7.49 mg kg^–1^). Green bars represent seedling rootstock families grafted with the CCN51 clonal scion in Cd-spiked soil (7.49 mg kg^–1^). Data are mean values ± SE (n = 8).

A decrease of 22.2% in total soluble protein content was observed in PA121 OP half-sib seedlings before grafting (Figure 12A). Two months after grafting, a significant decrease in total soluble protein content of 39.5 and 38.5% was observed respectively in the ICS95 clonal scion grafted on the IMC67 OP half-sib, and IMC67 **×** PA121 full-sib families, compared to the control plants without Cd addition (Figure 12B). A significant decrease in protein content in leaf tissue of the CCN51 clonal scion grafted on IMC67 OP half-sib progenies, equivalent to 31% compared with the control, was also observed 2 months after grafting (Figure 12C).

### Mineral Nutrients Content in Leaf Tissue

Cadmium absorption resulted in changes in the accumulation of mineral elements in leaf tissue of ungrafted seedling (Supplementary Table 1). Significant increases of 14 and 17.3% in K content were observed in PA121 OP half-sib and PA121 **×** IMC67 full-sib families compared with the control. N content decreased in all seedling families, while Mg content increased. However, these changes were significant only in IMC67 **×** PA121 full-sib progenies, compared with the control (without Cd addition). A significant increase in P content in leaf tissue was observed only in IMC67 **×** PA121 full-sib family established in Cd-spiked soil. In relation to micronutrients, changes in Fe content in leaf tissue were significant only in IMC67 OP half-sib progenies, for which there was a reduction of 16.3% in relation to the control. Zn and Mn content decreased in all seedling families subjected to stress by Cd. A significantly higher decrease in the Zn content of 25.8% was observed in PA121 OP half-sib progenies, compared with the control. Mn content respectively decreased by 10.9, 24.4, 11.5, and 21.5% for PA121 and IMC67 OP half-sib, and PA121 **×** IMC67 and IMC67 **×** PA121 full-sib families, in relation to the control.

Mineral elements concentration in leaf tissue of clonal scions across seedling rootstock families is shown in Supplementary Table 2. Two months after grafting, significant changes were observed in the concentrations of macro- and microelements in both CCN51 and ICS95.

### Rootstock-Mediated Specific Combining Abilities and Heritability Scores in Full-Sib Families

In ungrafted full-sib progenies obtained from the crossing between IMC67 **×** PA121 and its reciprocal cross, specific combining abilities were only significantly different for the Cd treatment (Supplementary Table 3). Specifically, they differed significantly in 23 of the 38 examined traits, including Cd content in roots, eight nutrient uptake traits, six biomass traits, three cationic interchange traits, and five pigments’ content traits (Table 1). Meanwhile, only two traits exhibited heritability (*h*^2^) scores above 0.1 under the non-Cd-added treatment, as compared with seven from the Cd-added treatment (Supplementary Table 4). Of the latter, all matched with significantly different specific combining abilities (Table 1), which speaks for a predominant role of additive genetic effects for ungrafted progenies.

**TABLE 1:** Specific combining abilities and narrow-sense heritability (*h*^2^) in cacao seedlings from controlled crosses before grafting.

Trait		MLM						*h* ^2^			
		F	*p*	IMC67	PA121	IMC67 × PA121	PA121 × IMC67	Median	2.5%	97.5%	*r*
Cadmium	Cd (mg kg^–1^)	1.80	0.174	85.20	–6.71	2.05	18.54	0.048	0.016	0.183	0.163
	Cd in root (mg kg^–1^)	3.35	**0.035**	40.36	11.59	–0.39	19.48	0.054	0.017	0.231	0.287
Nutrients	K (%)	5.56	**0.005**	1.57	0.15	–0.09	–0.01	0.068	0.022	0.316	0.410
	Mg (%)	5.33	**0.006**	0.48	–0.08	–0.04	–0.08	0.084	0.023	0.351	0.472
	Fe (mg kg^–1^)	17.05	**0.000**	175.50	–40.75	–3.63	7.00	**0.169**	0.034	0.660	0.651
	Mn (mg kg^–1^)	5.32	**0.006**	489.13	79.75	70.75	136.25	0.067	0.021	0.274	0.398
	Cu (mg kg^–1^)	4.16	**0.016**	10.31	1.37	–0.90	–0.26	0.057	0.017	0.233	0.308
	Zn (mg kg^–1^)	5.64	**0.004**	68.01	–15.61	–8.98	–3.56	0.090	0.024	0.350	0.504
	B (mg kg^–1^)	4.03	**0.018**	67.78	–19.04	–10.90	–14.35	0.088	0.023	0.327	0.505
	Na (mg kg^–1^)	8.09	**0.001**	177.63	–30.00	–26.88	–10.50	0.074	0.022	0.303	0.439
Biomass	Leaf fresh weight (g)	7.53	**0.001**	46.55	1.55	11.01	4.78	0.050	0.017	0.332	0.543
	Stem fresh weight (g)	16.53	**0.000**	34.95	–11.38	0.68	–0.61	**0.225**	0.046	0.816	0.705
	Aerial length (cm)	3.89	**0.021**	56.40	–7.15	–0.05	4.31	0.066	0.018	0.285	0.361
	Leaf area (cm^2^)	12.19	**0.000**	3217.31	–47.31	1018.14	488.41	0.057	0.016	0.377	0.601
	Dry stem weight (g)	14.50	**0.000**	9.26	–3.04	0.54	–0.09	**0.201**	0.035	0.695	0.675
	Dry root weight (g)	4.06	**0.018**	11.81	–3.68	0.81	–0.07	0.071	0.021	0.336	0.441
Leaf gas exchange	P_N_ (μmol CO2 m^–2^ s^–1^)	3.38	**0.034**	5.21	–0.04	1.61	3.01	0.049	0.016	0.190	0.310
	WUE_INST_ (μmol CO_2_ mmol H_2_O)	4.23	**0.015**	0.90	0.02	0.40	0.51	0.048	0.015	0.201	0.428
Electrolyte leakage	Electrolyte leakage (%)	3.10	**0.045**	23.93	4.17	–1.00	–3.89	0.056	0.018	0.245	0.298
Chloroplast pigments	Chlorophyll *A* (mg/g)	11.53	**0.000**	4.49	–1.62	–1.66	–0.97	**0.172**	0.041	0.648	0.647
	Chlorophyll *B* (mg/g)	11.42	**0.000**	1.65	–0.60	–0.59	–0.27	**0.151**	0.030	0.531	0.631
	Total chlorophyll (*a* + *b*) (mg/g)	11.52	**0.000**	6.14	–2.22	–2.25	–1.23	**0.162**	0.037	0.568	0.644
	Carotenoids (mg/g)	13.52	**0.000**	1.37	–0.48	–0.36	–0.36	**0.252**	0.064	0.686	0.740
Total soluble protein	Protein (mg/g)	5.85	**0.004**	0.57	–0.07	0.17	0.35	0.052	0.016	0.231	0.326

*Estimates were gathered in two OP half-sib families (IMC67 and PA121), and two full-sib progenies obtained from the crossing between IMC67 × PA121 and its reciprocal cross. Only significant (bold and underlined) specific combining abilities under the Cd-added treatment are retained, and only h^2^ estimates above 0.1 are highlighted, except for the Cd content in leaves that is kept for reference purposes. For the full compilation refer to Supplementary Tables 3, 4 for the specific combining abilities and the narrow-sense heritability, respectively. Specific combining abilities are indexed to a general mean (intercept under the first column after the p-value), and the corresponding positive or negative contributions (three last columns). Heritability (h^2^) and model fits (R software, R Core Team) estimates were computed using an A pedigree relatedness matrix (Supplementary Figure 2A) inputted in a “genetic prediction” additive mixed linear model, according to de los Campos et al. (2009).*

Concerning cacao full-sib seedlings from controlled crosses between IMC67 **×** PA121 (and reciprocal) 2-months after grafting with the ICS95 and CCN51 scions, specific combining abilities were mostly different under the Cd-added treatment (Supplementary Table 5). Specifically, these differences concerned 27 traits from the overall 35 examined traits (Table 2). Mismatches due to the scion origin were overserved in 14 cases (including Cd content in leaves and roots). Similarly, when examining the very same cacao seedlings from these controlled crosses 4 months after grafting, specific combining abilities differed for nine of the 17 studied traits (Supplementary Table 6). Specifically, these differences only overlapped between the ICS95 and CCN51 clonal scions for Cd content in leaves and dry root weight (Table 2).

**TABLE 2:** Specific combining abilities in cacao seedling rootstocks from controlled crosses 2 and 4 months after grafting with the ICS95 and CCN51 scions.

Sampling	Trait		ICS95						CCN51					
			*F*	*p*	IMC67	PA121	IMC67 × PA121	PA121 × IMC67	*F*	*p*	IMC67	PA121	IMC67 × PA121	PA121 × IMC67
Two months after grafting	Cadmium	Cd in leaf tissue (mg kg^–1^)	5.38	**0.006**	**81.39**	**14.29**	**19.40**	**29.58**	0.55	0.653	121.13	–17.61	–5.95	–3.44
		Cd in roots (mg kg^–1^)	0.26	0.856	33.65	0.76	–1.54	1.44	4.23	**0.016**	**37.37**	**–3.42**	**1.33**	**11.54**
	Nutrients	N (%)	5.31	**0.006**	**1.54**	**0.05**	**–0.04**	**0.27**	6.33	**0.002**	**1.67**	**0.06**	**–0.15**	**0.24**
		K (%)	5.69	**0.004**	**1.76**	**0.31**	**0.08**	**0.02**	6.55	**0.002**	**1.72**	**0.19**	**0.25**	**0.14**
		Mg (%)	3.17	**0.042**	**0.71**	**–0.11**	**–0.01**	**–0.09**	6.59	**0.002**	**0.75**	**–0.13**	**0.01**	**–0.17**
		Cu (mg kg^–1^)	3.04	**0.048**	**8.12**	**3.73**	**1.11**	**2.33**	6.87	**0.002**	**9.98**	**3.19**	**–1.61**	**2.40**
		Zn (mg kg^–1^)	3.74	**0.024**	**63.73**	**–14.80**	**4.51**	**8.70**	2.07	0.130	66.45	–13.83	0.04	–0.30
		Na (mg kg^–1^)	3.18	**0.041**	**113.10**	**12.53**	**21.28**	**–5.64**	2.66	0.070	107.49	–5.00	22.50	2.49
	Biomass	Root fresh weight (g)	9.12	**0.000**	**39.68**	**–12.74**	**1.51**	**1.33**	5.23	**0.006**	**36.39**	**–10.38**	**2.60**	**–0.59**
		Dry root weight (g)	11.08	**0.000**	**16.65**	**–5.88**	**1.16**	**–0.16**	4.71	**0.010**	**15.53**	**–4.58**	**1.05**	**–0.84**
	Chlorophyll fluorescence	Fm	3.03	**0.048**	**377.56**	**–7.56**	**–33.63**	**22.75**	1.51	0.238	370.50	–4.81	–12.38	29.88
		Fv/Fm	4.50	**0.012**	**0.77**	**–0.02**	**–0.02**	**0.01**	1.74	0.185	0.76	–0.03	–0.01	0.01
		Fv/Fo	4.93	**0.008**	**3.41**	**–0.32**	**–0.26**	**0.20**	1.34	0.284	3.11	–0.28	–0.09	0.19
	Chloroplast pigment	Chlorophyll *A* (mg/g)	4.01	**0.019**	**1.53**	**0.03**	**–0.35**	**0.25**	5.80	**0.004**	**1.44**	**0.11**	**–0.44**	**0.09**
		Chlorophyll *B* (mg/g)	4.44	**0.012**	**0.59**	**0.10**	**–0.11**	**0.12**	11.05	**0.000**	**0.64**	**0.58**	**–0.04**	**–0.04**
		Total chlorophyll (*a* + *b*) (mg/g)	4.13	**0.017**	**2.12**	**0.12**	**–0.46**	**0.37**	7.31	**0.001**	**2.08**	**0.69**	**–0.48**	**0.05**
		Carotenoids (mg/g)	5.31	**0.006**	**0.64**	**–0.04**	**–0.14**	**0.10**	4.41	**0.013**	**0.63**	**0.11**	**–0.13**	**0.03**
	Total soluble protein	Protein (mg/g)	13.87	**0.000**	**0.46**	**0.45**	**0.13**	**0.22**	7.04	**0.001**	**0.52**	**0.26**	**0.20**	**0.37**
Four months after grafting	Cadmium	Cd in leaf tissue (mg kg^–1^)	3.96	**0.021**	**66.97**	**6.70**	**16.73**	**40.48**	4.94	**0.009**	**84.10**	**–5.19**	**33.07**	**35.24**
		Cd in roots (mg kg^–1^)	7.08	**0.002**	**35.45**	**21.24**	**–4.21**	**21.30**	0.33	0.803	50.55	–5.33	–8.64	–7.79
	Biomass	Leaf fresh weight (g)	1.76	0.183	69.38	–7.30	–3.18	3.45	7.65	**0.001**	**61.61**	**–2.03**	**–8.83**	**11.96**
		Root weight (g)	3.98	**0.020**	**41.34**	**–4.64**	**8.91**	**7.70**	2.30	0.102	46.11	–7.96	5.16	0.24
		Leaf area (cm^2^)	1.34	0.286	4022.02	–324.64	–74.60	293.20	3.50	**0.030**	**3925.93**	**–100.12**	**–121.21**	**843.41**
		Dry leaf weight (g)	2.37	0.097	20.85	–2.95	0.55	2.54	7.74	**0.001**	**18.22**	**0.28**	**–1.66**	**5.19**
		Dry root weight (g)	5.48	**0.005**	**14.68**	**–2.00**	**4.89**	**3.51**	4.93	**0.008**	**16.61**	**–2.56**	**6.30**	**0.60**
	Chlorophyll fluorescence	Fv/Fm	3.30	**0.038**	**0.78**	**–0.01**	**–0.02**	**0.01**	1.06	0.383	0.77	–0.05	0.01	–0.02
		Fv/Fo	3.19	**0.043**	**3.59**	**–0.14**	**–0.35**	**0.29**	1.37	0.275	3.46	–0.34	0.20	–0.31

*Estimates were gathered in two OP half-sib families (IMC67 and PA121), and two full-sib progenies obtained from the crossing between IMC67 × PA121 and its reciprocal cross, grafted with the ICS95 and CCN51 clonal scions. Only significant (bold and underlined) specific combining abilities under the Cd-added treatment are retained. For the full compilation refer to Supplementary Tables 5, 6 for specific combining abilities 2 and 4 months after grafting, respectively. Specific combining abilities are indexed to a general mean (intercept under the first column after the p-value), and the corresponding positive or negative contributions (three last columns).*

Most of these specific combining abilities from the full-sib rootstock families fell when looking at the narrow-sense rootstock-mediated heritability (*h*^2^) scores 2 months after grafting (Supplementary Table 7), and completely vanished after 4 months (Supplementary Table 8). When grafting the clonal scion ICS95, heritability scores above 0.1 were only observed 2 months after grafting for root weight, dry root weight, and protein content, whereas for the CCN51 scion this only applied for chlorophyll *b* (Table 3). Four-months after grafting, none *h*^2^ estimate was above 0.1.

**TABLE 3:** Narrow-sense rootstock-mediated heritability (*h*^2^) in cacao seedling rootstocks from controlled crosses 2 months after grafting with the ICS95 and CCN51 scions.

Trait		ICS95				CCN51			
		Median	2.5%	97.5%	*r*	Median	2.5%	97.5%	*r*
Cadmium	Cd in leaf tissue (mg kg^–1^)	0.056	0.018	0.232	0.289	0.052	0.017	0.209	0.200
	Cd in roots (mg kg^–1^)	0.049	0.015	0.196	0.058	0.052	0.017	0.236	0.231
Biomass	Root fresh weight (g)	**0.125**	**0.028**	**0.580**	**0.584**	0.079	0.020	0.325	0.446
	Dry root weight (g)	**0.146**	**0.033**	**0.660**	**0.612**	0.068	0.019	0.289	0.406
Chloroplast pigment	Chlorophyll B (mg/g)	0.054	0.018	0.237	0.267	**0.149**	**0.032**	**0.706**	**0.631**
Total soluble protein	Protein (mg/g)	**0.274**	**0.077**	**0.660**	**0.760**	0.079	0.022	0.362	0.475

*Estimates were gathered in two OP half-sib families (IMC67 and PA121), and two full-sib progenies obtained from the crossing between IMC67 × PA121 and its reciprocal cross, grafted with the ICS95 and CCN51 clonal scions. Only h^2^ estimates above 0.1 (highlighted) under the Cd-added treatment are retained, except for the Cd content in leaves and roots that is kept for reference purposes (for the full compilation refer to Supplementary Tables 7, 8 for 4-months after grafting). Heritability (h^2^) and model fits (r) estimates were computed using an A pedigree relatedness matrix (Supplementary Figure 2B) inputted in a “genetic prediction” additive mixed linear model according to de los Campos et al. (2009).*

## Discussion

### Cadmium Toxicity Affects a Wide Spectrum of Growth and Physiological Traits Before and After Grafting

In this study, an inhibition in plant growth and biomass was observed as result of Cd toxicity. Cd showed a greater toxic effect on the growth of PA121 OP seedlings than in the other families used as rootstocks. Also, a significant decrease in root biomass of PA121 OP rootstocks was observed both in combinations with clones ICS95 and CCN51 as scions. An effect of toxic levels of Cd on decreasing plant growth and biomass accumulation has been reported for other plant species such as cotton (Farooq et al., 2016), mustard (Ahmad et al., 2011), peanut (Lu et al., 2013), lettuce (Dias et al., 2012), lupin (Zornoza et al., 2002), and *Rorippa globose* (Wei et al., 2012), but this is the first explicit quantification in cacao scion × rootstocks combinations. Two months after grafting, changes were observed in growth parameters, although these were significant only for root biomass. Interestingly, 4 months after grafting, rootstock families having ICS95 as scion continued exhibiting significant changes only for root biomass, whereas the rootstock families having CCN51 as scion already started presenting significant changes also for growth parameters measured on the aerial part such as leaf area and leaf biomass. Decreased leaf area may be the result of reduction in cells size or alternatively more condense intercellular spaces (Barceló et al., 1988). Such effect of Cd on plant growth may result from alterations in cell division (Liu et al., 2003).

Meanwhile, a reduced Cd translocation from root to shoot could be explained by a differential loading of Cd into the xylem (Arao et al., 2010). Differences in Cd uptake by a symplastic pathway could also be related to differences between genotypes in Cd translocation to the stem of rootstocks and to scion’s tissues (Arao et al., 2010). The latter would explain a lower accumulation of Cd in ICS95, grafted on IMC67, compared with CCN51 grafted on the same rootstock. Expression of specific transporter proteins could also explain differences in Cd uptake and accumulation in different cacao genotypes (Moore et al., 2020). Still, further studies are needed to evaluate Cd translocation in the xylem of rootstocks × scion combinations to increase knowledge of Cd distribution in plant organs.

Cadmium has an effect on different photosynthetic processes such as decrease in chlorophyll content, inhibition of chlorophyll formation, inhibition of Rubisco activity, inhibition of both photosynthesis reaction centers, PSI and PSII, and an increase in lipoxygenase activity (Rai et al., 2016). The inhibition of the growth of plants subjected to Cd stress could further be due to a toxic effect of the heavy metal on physiological processes, such as reduction of the maximum photochemical efficiency of PSII (Ge et al., 2015; Pereira de Araújo et al., 2017), in addition to an imbalance in the uptake of essential mineral elements (Zornoza et al., 2002; Hédiji et al., 2015) and to an inhibition in the production of sugars due to the decrease in the carbon assimilation rate, P_N_ (Dias et al., 2012). Ahmad et al. (2011) correlated the toxic effect of Cd on mustard plant growth with a Cd-induced decrease in P_N_ and WUE. Similarly, a greater decreasing effect of Cd concentration in P_N_ and WUE_INST_ was observed in our study for PA121 that, as indicated above, was the rootstock that presented a greater effect of Cd toxicity on plant growth. A decrease in WUE suggests alterations in water balance, which could be due to changes in water uptake and transport as a result of the toxic effect of Cd (Singh and Tewari, 2003).

Cadmium binds to several sites in PSII, affecting both the donor and the acceptor side. Cd also inhibits oxygen evolution in a high affinity site by competition with Ca on the donor side (Sigfridsson et al., 2004). Concerning underlying physiological processes, efficiency of PSII, measured as the fluorescence F_v_/F_m_ ratio, did not appear to be affected in Cd-stressed rootstocks. However, significant changes in F_v_/F_m_ ratio of scion ICS95 were observed at 2 and 4 months after grafting on IMC67 **×** PA121 full-sib family rootstocks established in Cd-spiked soil compared with the control. Interestingly, no significant differences were observed in the maximum efficiency of the reaction centers of the PSII of scion CCN51 grafted on any of the rootstocks subjected to Cd stress in soil. This result suggested that grafting combinations having CCN51 as scion had a more stable photosynthetic performance than those having ICS95 clone as scion. A similar effect of Cd on photosynthetic performance has also been reported in poplar (Ge et al., 2015; Jiao et al., 2015), pea (Sandalio et al., 2001), and lettuce (Dias et al., 2012). One of the factors that could result in an effect on photosynthetic performance is the decrease in chlorophyll content as a result of Cd toxicity (Sandalio et al., 2001). However, for the case of ungrafted rootstocks, an increase in the content of chloroplast pigments at IMC67 OP seedlings is observed and does not result in significant changes in the F_v_/F_m_ ratio. On the other hand, changes in the photosynthetic performance of scion ICS95 2 months after grafting effectively coincided with a decrease in chlorophyll content. In the case of scion CCN51, although a decrease in chlorophyll content is observed under Cd stress, the photosynthetic performance remains unchanged.

Similarly, a significant decrease in the concentration of chloroplast pigments has been reported for other species such as cotton (Farooq et al., 2016), beans (Saidi et al., 2013), *Phragmites australis* (Pietrini et al., 2003), poplar (Jiao et al., 2015), maize (Ekmekçi et al., 2008), pea (Sandalio et al., 2001), and cucumber plants exposed to Cd (Zhang et al., 2002). In this regard, carotenoids play a role as antioxidants, and increases in carotenoid content in some species have been explained as an attempt to protect chlorophyll from the photooxidative damage caused by Cd stress (Mishra et al., 2006). Therefore, the decrease in carotenoid content observed in grafting combinations having ICS95 as scion may have jeopardized the detoxification of radicals formed in response to Cd stress (Ekmekçi et al., 2008).

Cadmium absorption by plants can also result in changes in the accumulation of essential mineral elements in plant tissues (Castro et al., 2015; Pereira de Araújo et al., 2017). Cd may affect the transport of mineral elements by disturbing the radial movement of transporters in the root, loading into the xylem vessels or into the leaves, thereby promoting morphological changes of the xylem tissue, changes in H^+^-ATPase activity, and alterations in IRT1 transporter selectivity (Sandalio et al., 2001). Changes in nutritional elements may indicate alterations in ionic homeostasis (Saidi et al., 2013). In our work, changes in mineral elements were observed in rootstock families before grafting, and in both ICS95 and CCN51 leaf tissue 2 months after grafting. Cd translocation in the plant is carried out using the same transporters than some nutritional elements such as Ca, Mn, and Zn (Rahman et al., 2016). Competition for these same transporters may explain the decrease in the content of Zn and Mn in ungrafted rootstocks under Cd stress.

On the other hand, according to Küpper et al. (1998), Cd could replace Mg in chlorophylls, which may decrease chlorophyll content in plants under Cd stress. A direct proportional relationship between toxicity of heavy metals and Mg substitution in chlorophylls has been reported (Küpper et al., 1996). In our study, a significant increase in Mg concentration in leaf tissue was observed 2 months after grafting in Cd-spiked soil. These results are in agreement with the observation made by Ciećko et al. (2005), in which the content of Mg in oat increased when it was grown in Cd-contaminated soil. An effect of Cd on electron transport on the reducing side of photosystem I was observed on isolated chloroplasts of maize plants grown in nutrient solution containing the heavy metal. A reduction in electron transport was associated with a decrease in ferredoxin content, which was then correlated with a low Fe concentration, suggesting that Cd induced Fe deficiency (Siedlecka and Baszyński, 1993). In our work, significant changes in Fe content in leaf tissue of ungrafted rootstocks were observed only for IMC67 seedlings. Accordingly, a decrease in F_v_/F_m_ ratio scores was observed in IMC67 seedlings before grafting. A significant decrease in Fe content has also been observed in bean plants (Saidi et al., 2013). On the other hand, a significant increase in Fe content for cacao in grafting combinations having full-sib IMC67 **×** PA121 progenies as rootstocks was observed too. Despite this, a significant decrease in F_v_/F_m_ was observed in the ICS95 clone when grafted on this rootstock family, whereas no significant changes were observed in this parameter when using CCN51 as scion.

A decrease in the content of total soluble protein was observed in PA121 seedlings before grafting. Significant decreases were also observed in both CCN51 and ICS95 scions 2 months after grafting on the IMC67 OP rootstock family. An effect in total soluble protein content could be explained by Cd-induced protein degradation and an increase in proteolytic activity (Mishra et al., 2006). In some cases, a slight boost in total soluble protein was observed, but it was not significant compared with the control. Slight increases in protein content may be explained by the induction of stress proteins as part of the plant defense system to Cd toxicity (Di Toppi and Gabbrielli, 1999).

Finally, a significant increase in electrolytes leakage was observed in IMC67 and PA121 established in Cd-spiked soil, suggesting a Cd effect on cell membrane integrity. An increase in electrolyte loss has been observed in cucumber (Gonçalves et al., 2007), maize (Ekmekçi et al., 2008), and *Bacopa monnieri* (Mishra et al., 2006). Heavy metals induce an alteration in the lipid composition of thylakoid membranes. Reactive oxygen species (ROS) induced by Cd results in lipid peroxidation, which implies the degradation of polyunsaturated fatty acids of membrane lipids. The later causes distortion of the lipid bilayer and alters membrane ion channels, resulting in leakage of ions (Mishra et al., 2006). The apparent lack of cell membrane damages in the CCN51 and ICS95 scions, 2 months after grafting, may be due to an insufficient exposure time to Cd as to cause a perceptible effect.

### Rootstock Versus Scion Genetic Conflict

This work has enlightened some major trends regarding the complexity at the rootstock–scion interface. First, phenotypic differences due to rootstock effects are more notorious early after grafting (i.e., 2-months), in concert with expectations observed at the ungrafted seedlings. Such differences tend to vanish at older grafted seedlings (i.e., 4 months after grafting). The tendencies observed when computing specific combining abilities across rootstock families are aligned with this conclusion, in the sense that significantly different specific combining abilities among rootstock families were more common at 2 months after grafting than at 4 months. Second, heritability estimates also speak for the dilution of the rootstock effects through genotypes and time. Several of the heritabilities that were calculated in ungrafted families were not significant in the grafted portion of the experiment, which may speak for a genuine physiological communication gap at the graft interface. Alternatively, it could reflect an intrinsic limitation in degrees of freedom due to the complexity of the rootstock × scion factorial design. Still, rootstock-mediated heritability scores above 0.1 were more commonly observed at 2 months after grafting than at 4 months, regardless of the specific rootstock **×** scion combination. Such instability ultimately suggests an underlying and unavoidable conflict between the two genomes that shape the chimeric grafted organism (Warschefsky et al., 2016; Gautier et al., 2019).

Genetic conflict is known to be pervasive at multiple nested evolutionary scales, for example, among genes, among chromosomes, between chromosomes and cytoplasmic organelles, and between sexes in dioicous species (Ågren and Clark, 2018). These multiple scenarios are evidenced by the recurrent segregation distortion due to gene drives, transposons, and unconventional sex determination systems (Renner and Müller, 2021). However, our study is the first in suggesting an analogous mechanism at the rootstock **×** scion interface, typically regarded as leading to emergent heterotic properties (Reyes-Herrera et al., 2020). This conflict may be due to underlying additive and combined physiological drivers (Loupit and Cookson, 2020), such as water and nutrients uptake and transport, hormone production and transport, and large-scale movement of molecules during grafting and through time (Rasool et al., 2020).

### Foreseeing the Complexity of the Rootstock × Scion Interaction

The novelty of this work lays on the explicit comparison of rootstock families in terms of Cd uptake. However, after quantifying an additive component such as the inheritance of rootstock effects (i.e., rootstock-mediated genetic variance) across recombinant cacao saplings, a next step is to consider more thoroughly the complexity of the rootstock × scion interaction. After all, rootstock metabolites transcend the root system and could reach the grafted scion, i.e., rootstock’s additive contribution (Loupit and Cookson, 2020; Rasool et al., 2020), which in turn may have contrasting consequences on rootstock traits, i.e., scion’s additive contribution (Shu et al., 2017). These concurrent effects would ultimately feedback an emergent rootstock × scion interaction, a statistical interaction in the strict sense.

To be able to accurately estimate the rootstock × scion component, it will be necessary to further validate the rootstock-mediated pedigree-estimated heritability scores *via* controlled experiments across an expanded panel of clonal scion genotypes grafted on rootstocks with a more continuous gradient of pairwise relatedness values (Reyes-Herrera et al., 2020). Specifically, future experimental assessments might rely on factorial designs of diverse clonal scions grafted on clonal rootstocks. This would allow to reduce recombination uncertainty and optimize statistical power to estimate the interaction term.

From an analytical point of view, the *A*-BLUP model implemented here would be capable to condition pedigree-based rootstock-mediated heritability scores as a function of the scion’s pedigree. Alternatively, the rootstock × scion interaction may also be quantified *via* indirect genetic effect (IGE) models (Bijma, 2010, 2013; Fisher and Mcadam, 2019) at relatively low phenotyping costs. It would also be desirable to extend IGE monitoring through time as a way to validate whether some of the significant rootstock × scion effects may persist in adult trees, even several years after grafting.

### Perspectives

A recurrent caveat of pedigree-based heritability estimates concerns the potential fortuitous unbalanced between each phenotypic vector and the *A* pedigree-based relatedness matrix within the “genetic prediction” model. However, we did not detect this trend for any of the significantly rootstock-inherited traits. On the contrary, after examining for 2 years, as part of a parallel experiment, eight traits in full-sib families obtained by cross pollination of clonal accessions from the Cacao Germplasm at greenhouse conditions and two water regimes, we have been able to identify three candidate families for further testing. We recommend using this promissory dataset as reference (i.e., training) population to calibrate explicit eco-physiological mechanistic models (Loìpez-Hernández and Cortés, 2019) and last-generation machine learning algorithms (Cortés and López-Hernández, 2021; Montesinos-Loìpez et al., 2021) as innovative alternatives beyond *A*-BLUP models (Guevara-Escudero et al., 2021). As part of this task, we envision the following pipeline: (1) developing explicit eco-physiological indices for cacao targeting neo-tropical localities, (2) calibrating last-generation predictive breeding models aiming to forecast such indices based on extensive genealogical information, (3) extending the previous models to account for the complexity of the rootstock-scion interaction (i.e., G × S × E term, as expanded in the previous section), and (4) validating the corresponding predictions across seed orchards and cacao saplings at local nurseries to leverage natural variation for early selection (i.e., before grafting) of low Cd uptake. Ultimately, this combined strategy promises speeding up breeding of polygenic trait variation in a perennial tree crop, while accounting for the interaction of multiple genotypes at the rootstock–scion interface. In parallel, high throughput genotyping of the rootstock families will enable a more accurate description of the underlying genetic architecture.

## Data Availability Statement

The original contributions presented in the study are included in the article/Supplementary Material, further inquiries can be directed to the corresponding author.

## Author Contributions

CR-M conceived the original sampling and experiment and compiled the datasets. JF-P, CH-V, and CR-M collected phenotypic data at the greenhouses. JF-P and CH-V carried out lab work procedures. CH-V, JF-P, and AC prepared input datasets for statistical softwares and carried out data analyses. CR-M and AC drafted a first version of this manuscript and edited by the other co-authors. CH-V, JF-P, AC, MM-D-T, CR-M, and VB interpreted results, contributed to the manuscript, and approved the submitted version.

## Author Disclaimer

The findings and conclusion in this publication are those of the authors and should not be construed to represent any official USDA or United States Government determination or policy.

## Conflict of Interest

The authors declare that the research was conducted in the absence of any commercial or financial relationships that could be construed as a potential conflict of interest.

## Publisher’s Note

All claims expressed in this article are solely those of the authors and do not necessarily represent those of their affiliated organizations, or those of the publisher, the editors and the reviewers. Any product that may be evaluated in this article, or claim that may be made by its manufacturer, is not guaranteed or endorsed by the publisher.

## Supplementary Material

Supplementary Figure 1 Pearson correlations coefficients among examined traits from half-sib and full-sib seedling rootstocks **(A)** before grafting, **(B)** two, and **(C)** four months after grafting. Estimates were gathered in two OP half-sib families (IMC67 and PA121), and two full-sib rootstock progenies obtained from the crossing between IMC67 × PA12 and its reciprocal cross, grafted with the ICS95 and CCN51 clonal scions under two Cd treatments. Correlation estimates are presented above the diagonal and below diagonal circles are sized and coloured accordingly.

Supplementary Figure 2 Heat plots of A pedigree relatedness matrices from two OP half-sib families (IMC67 and PA121), and two full-sib rootstock families derived from controlled crosses between IMC67 × PA121 and its reciprocal cross, inputed in a “genetic prediction” additive mixed linear model, according to de los Campos et al. (2009). Heat plots are depicted for verification purposes **(A)** before grafting, and **(B)** two and **(C)** four months after grafting with the ICS95 and CCN51 clonal scions under two Cd treatments.

Supplementary Table 1 Concentration of mineral nutrients in leaf tissue of ungrafted rootstocks five months after cadmium treatment. Different lowercase letters indicate significant differences in mineral elements concentration between ungrafted seedling families at the same Cd concentration in soil. Different capital letters indicate significant differences in mineral elements concentration of the same ungrafted seedling at different Cd concentrations in soil. Results are according to Duncan test (*p* < 0.05). Data are mean values ± SE (*n* = 8).

Supplementary Table 2 Concentration of mineral nutrients in leaf tissue of scions ICS95 and CCN51 two months after grafting onto rootstocks. Different lowercase letters indicate significant differences in mineral elements concentration in leaf tissue of the ICS95 or CCN5 clonal scions grafted on four different seedling rootstock families at the same Cd concentration in soil. Different capital letters indicate significant differences in mineral elements concentration in leaf tissue of ICS95 or CCN51 clonal scions grafted on the same seedling rootstock family at different Cd concentrations in soil. Results are according to Duncan test (*p* < 0.05). Data are mean values ± SE (*n* = 8).

Supplementary Table 3 Specific combining abilities in cacao seedlings from controlled crosses before grafting. Estimates were gathered in two OP half-sib families (IMC67 and PA121), and two full-sib progenies obtained from the crossing between IMC67 × PA121 and its reciprocal cross, under two Cd treatments. Specific combining abilities are indexed to a general mean (intercept under the first column after the *p*-value), and the corresponding positive or negative contributions (three last columns). Significantly different specific combining abilities are highlighted.

Supplementary Table 4 Narrow-sense heritability (h2) in cacao seedlings from controlled crosses before grafting. Estimates were gathered in two OP half-sib families (IMC67 and PA121), and two full-sib progenies obtained from the crossing between IMC67 × PA121 and its reciprocal cross, grafted with the ICS95 and CCN51 clonal scions under two Cd treatments. Heritability (*h*^2^) and model fits (*r*) estimates were computed using an *A* pedigree relatedness matrix (Supplementary Figure 2A) inputted in a “genetic prediction” additive mixed linear model, according to de los Campos et al. (2009). Heritability (*h*^2^) scores above 0.1 are highlighted.

Supplementary Table 5 Specific combining abilities in cacao seedling rootstocks from controlled crosses two-months after grafting. Estimates were gathered in full-sib rootstock progenies obtained from the crossing between IMC67 × PA121, and its reciprocal cross, grafted with the ICS95 and CCN51 clonal scions under two Cd treatments. Specific combining abilities are indexed to a general mean (intercept under the first column after the *p*-value), and the corresponding positive or negative contributions (three last columns). Significantly different specific combining abilities are highlighted.

Supplementary Table 6 Specific combining abilities in cacao seedling rootstocks from controlled crosses four-months after grafting. Estimates were gathered in two OP half-sib families (IMC67 and PA121), and two full-sib progenies obtained from the crossing between IMC67 × PA121 and its reciprocal cross, grafted with the ICS95 and CCN51 clonal scions under two Cd treatments. Specific combining abilities are indexed to a general mean (intercept under the first column after the *p*-value), and the corresponding positive or negative contributions (three last columns). Significantly different specific combining abilities are highlighted.

Supplementary Table 7 Narrow-sense rootstock-mediated heritability (h2) in cacao seedling rootstocks from controlled-crosses two-months after grafting. Estimates were gathered in two OP half-sib families (IMC67 and PA121), and two full-sib progenies obtained from the crossing between IMC67 × PA121 and its reciprocal cross, grafted with the ICS95 and CCN51 clonal scions under two Cd treatments. Heritability (*h*^2^) and model fits (*r*) estimates were computed using an *A* pedigree relatedness matrix (Supplementary Figure 2B) inputted in a “genetic prediction” additive mixed linear model, according to de los Campos et al. (2009). Heritability (*h*^2^) scores above 0.1 are highlighted.

Supplementary Table 8 Narrow-sense rootstock-mediated heritability (h2) in cacao seedling rootstocks from controlled-crosses four-months after grafting. Estimates were gathered in two OP half-sib families (IMC67 and PA121), and two full-sib progenies obtained from the crossing between IMC67 × PA121 and its reciprocal cross, grafted with the ICS95 and CCN51 clonal scions under two Cd treatments. Heritability (*h*^2^) and model fits (*r*) estimates were computed using an *A* pedigree relatedness matrix (Supplementary Figure 2C) inputted in a “genetic prediction” additive mixed linear model, according to de los Campos et al. (2009). None *h*^2^ estimate was above 0.1.
