# Supplementary figures and images for: Rootstock-Mediated Genetic Variance in Cadmium Uptake by Juvenile Cacao (Theobroma cacao L.) Genotypes, and Its Effect on Growth and Physiology
Source: Front Plant Sci. 2021 Dec 23;12:777842. doi: 10.3389/fpls.2021.777842 (PMC8733334; doi:10.3389/fpls.2021.777842)

A

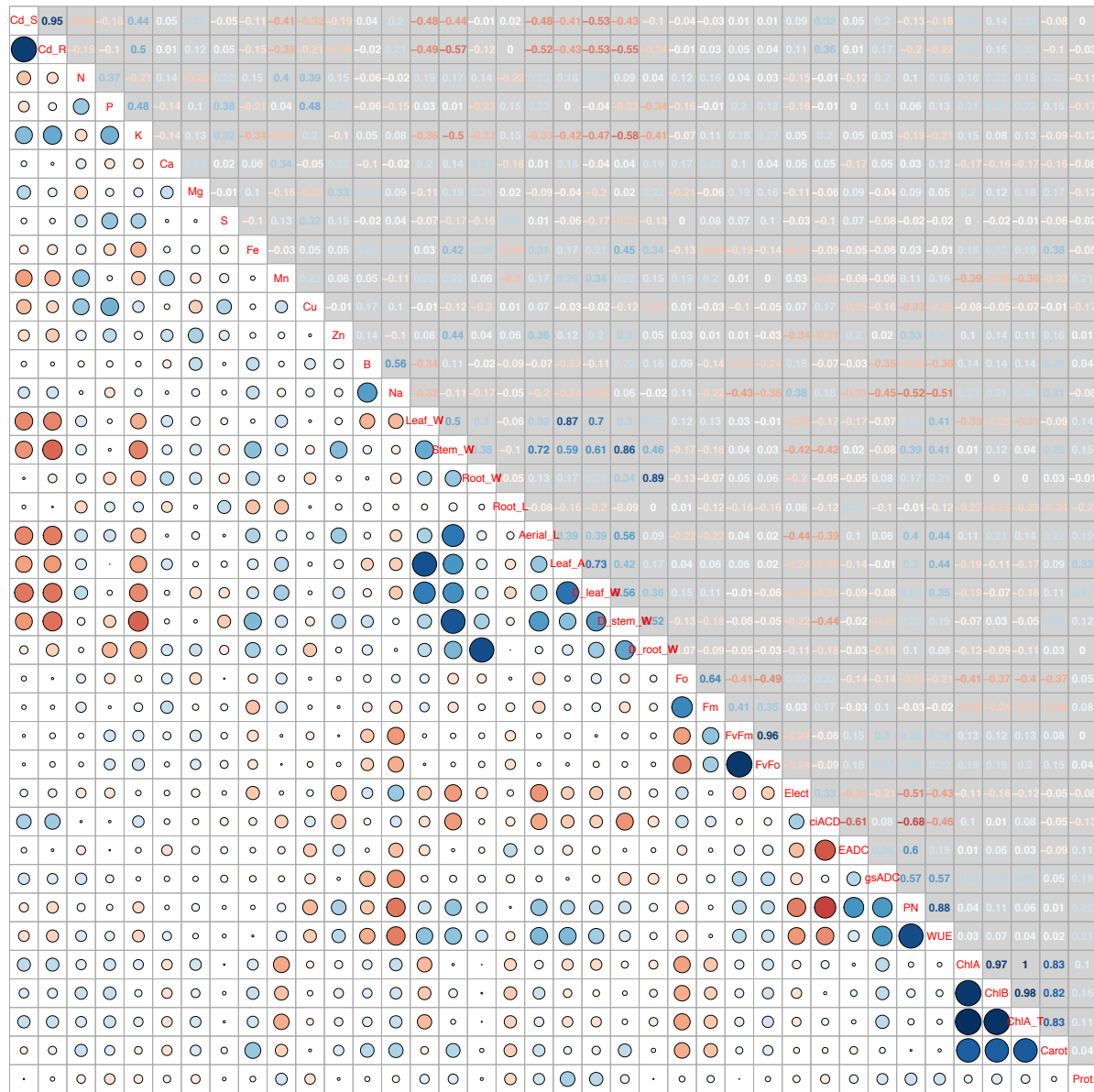

B

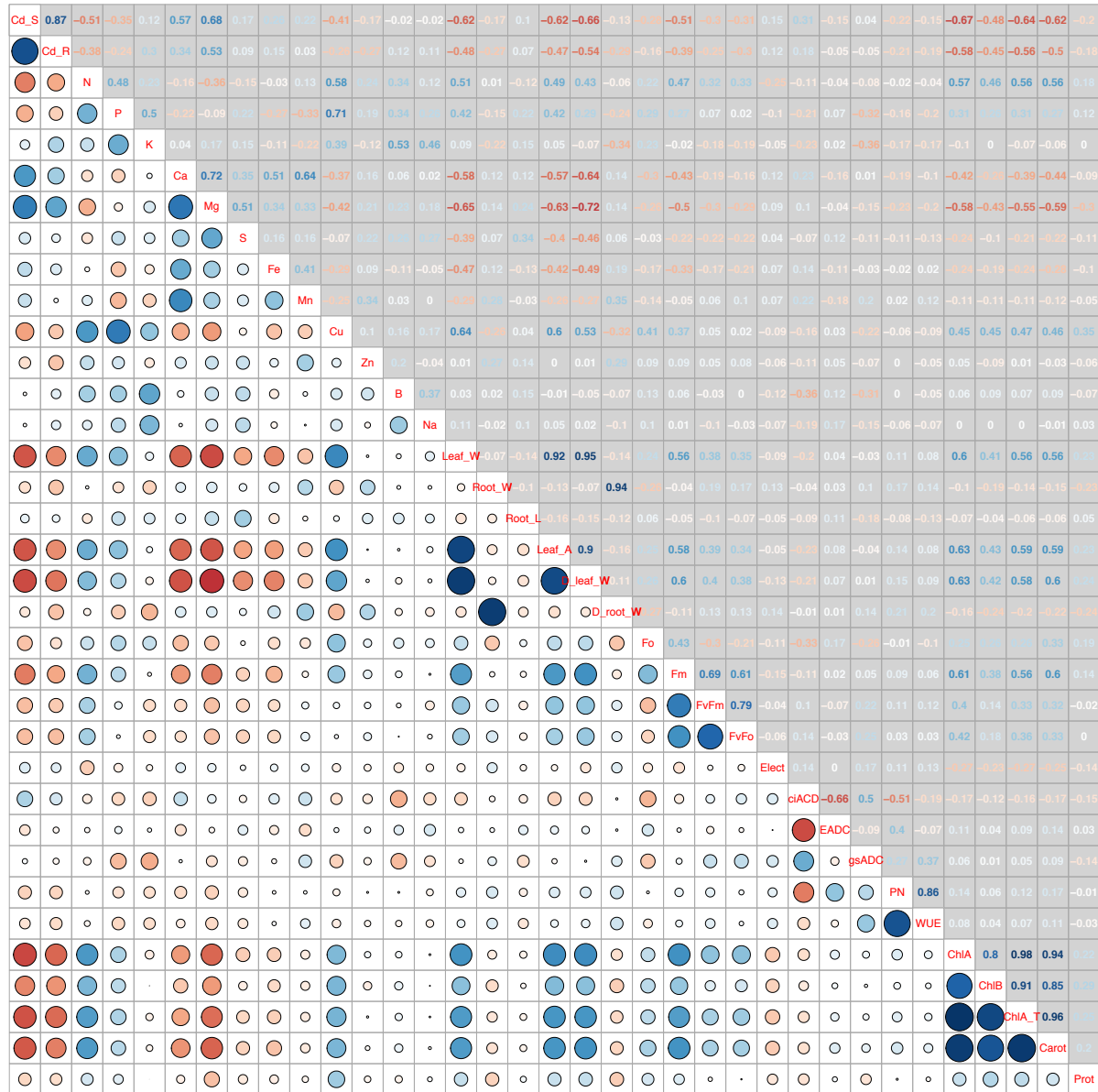

C

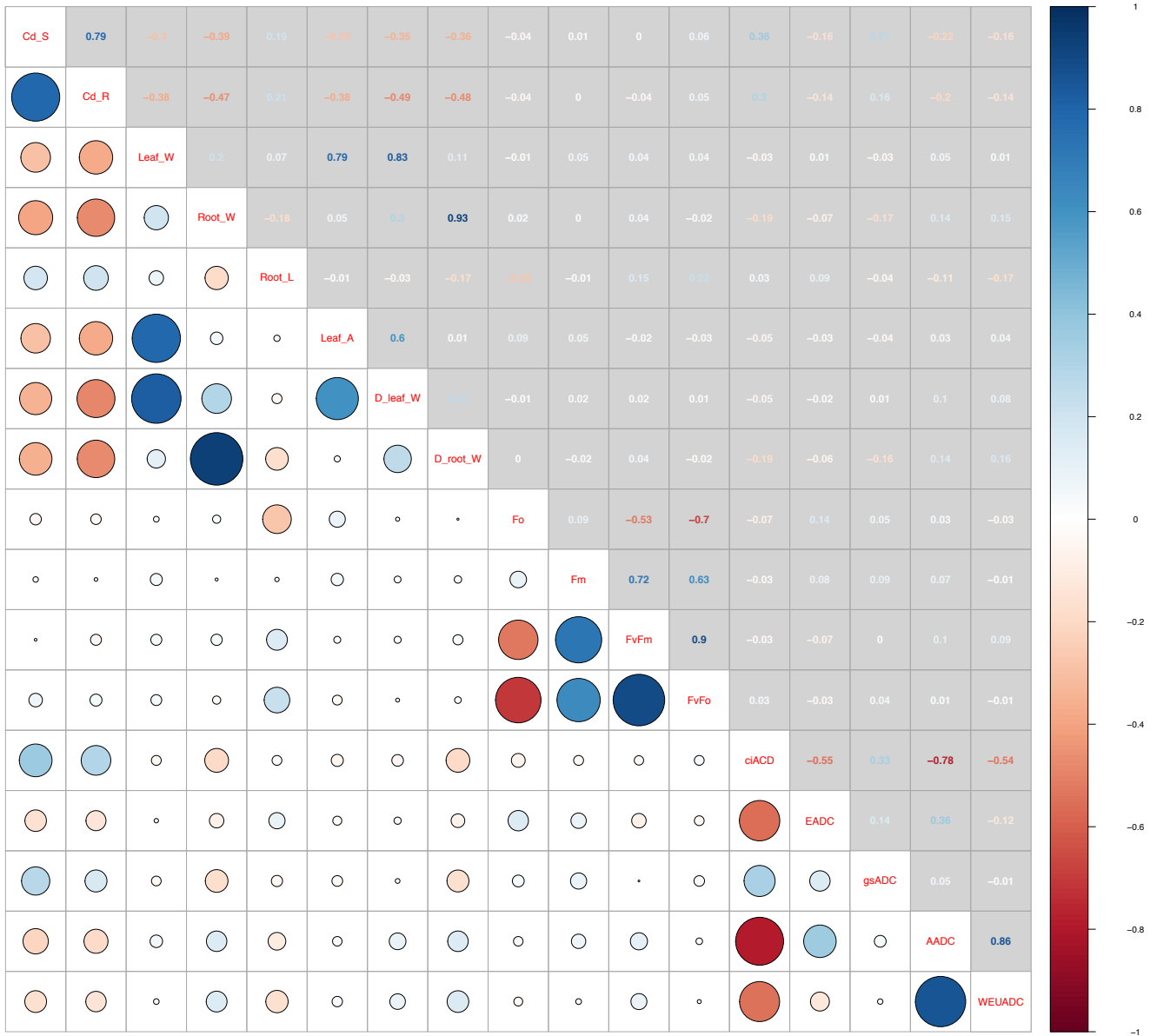

Supplement: Supplementary Figure 1 — Pearson correlations coefficients among examined traits from half-sib and full-sib seedling rootstocks (A) before grafting, (B) two, and (C) four months after grafting. Estimates were gathered in two OP half-sib families (IMC67 and PA121), and two full-sib rootstock progenies obtained from the crossing between IMC67 × PA12 and its reciprocal cross, grafted with the ICS95 and CCN51 clonal scions under two Cd treatments. Correlation estimates are presented above the diagonal and below diagonal circles are sized and coloured accordingly. [file Image_1.pdf]

A

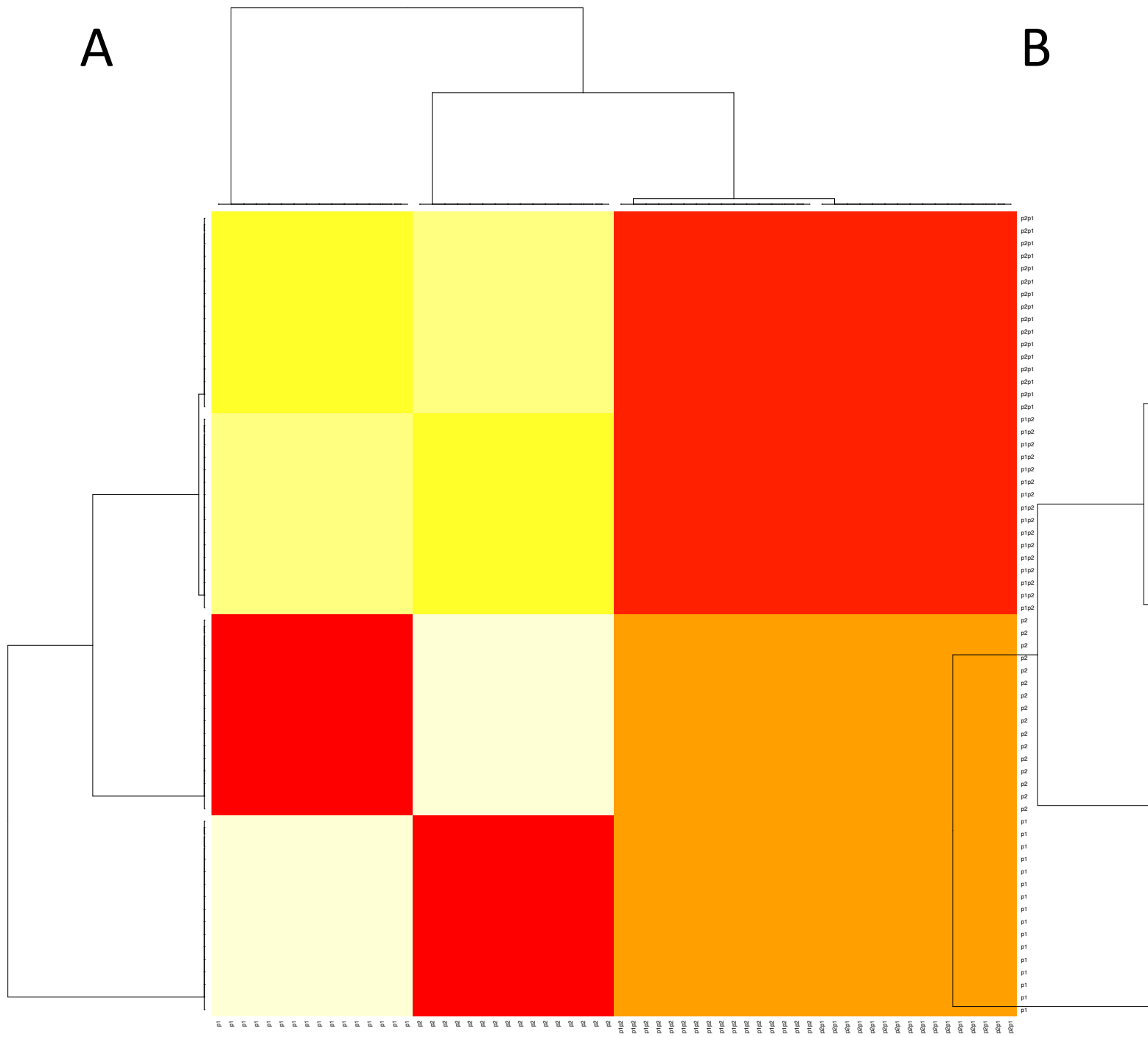

B

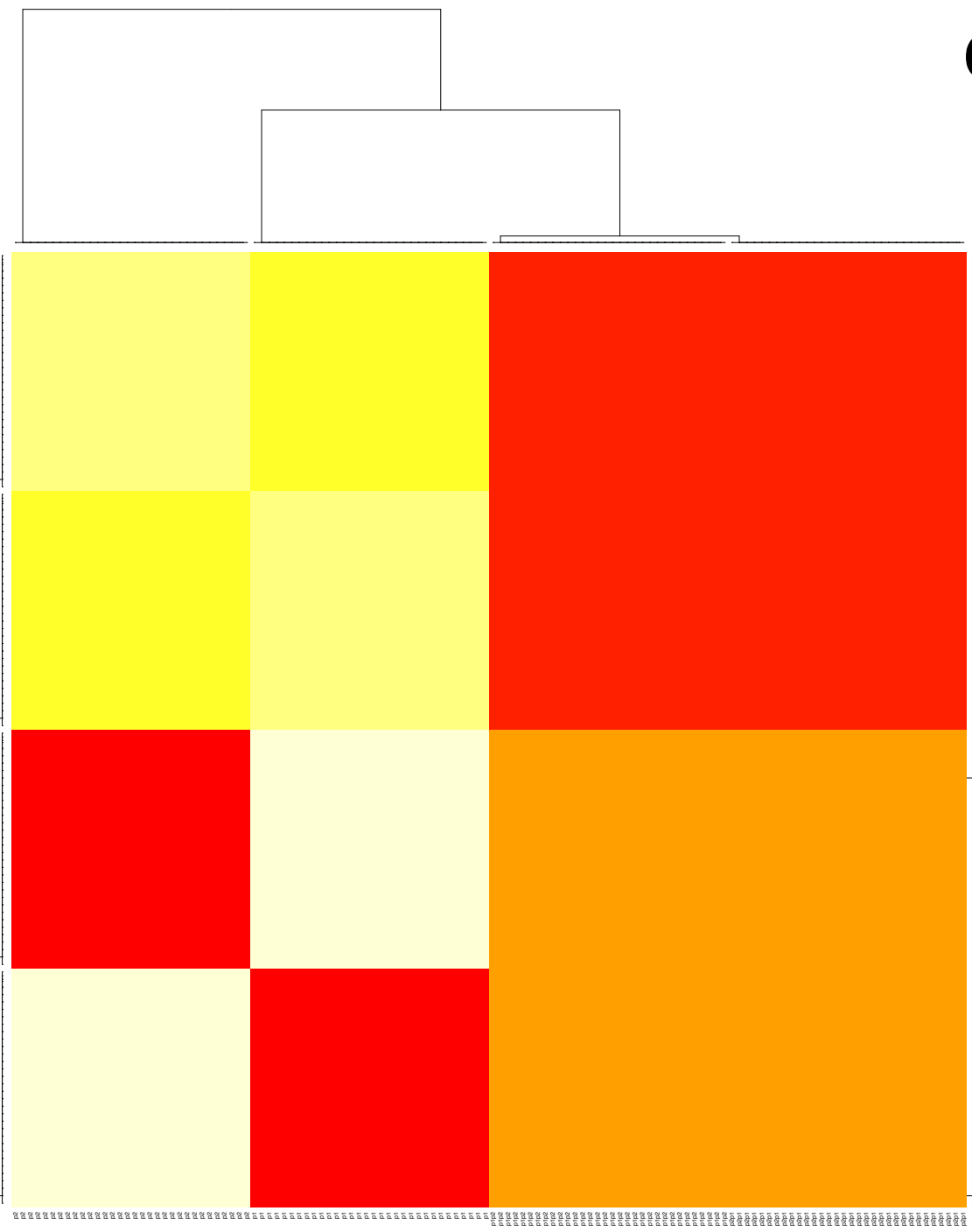

C

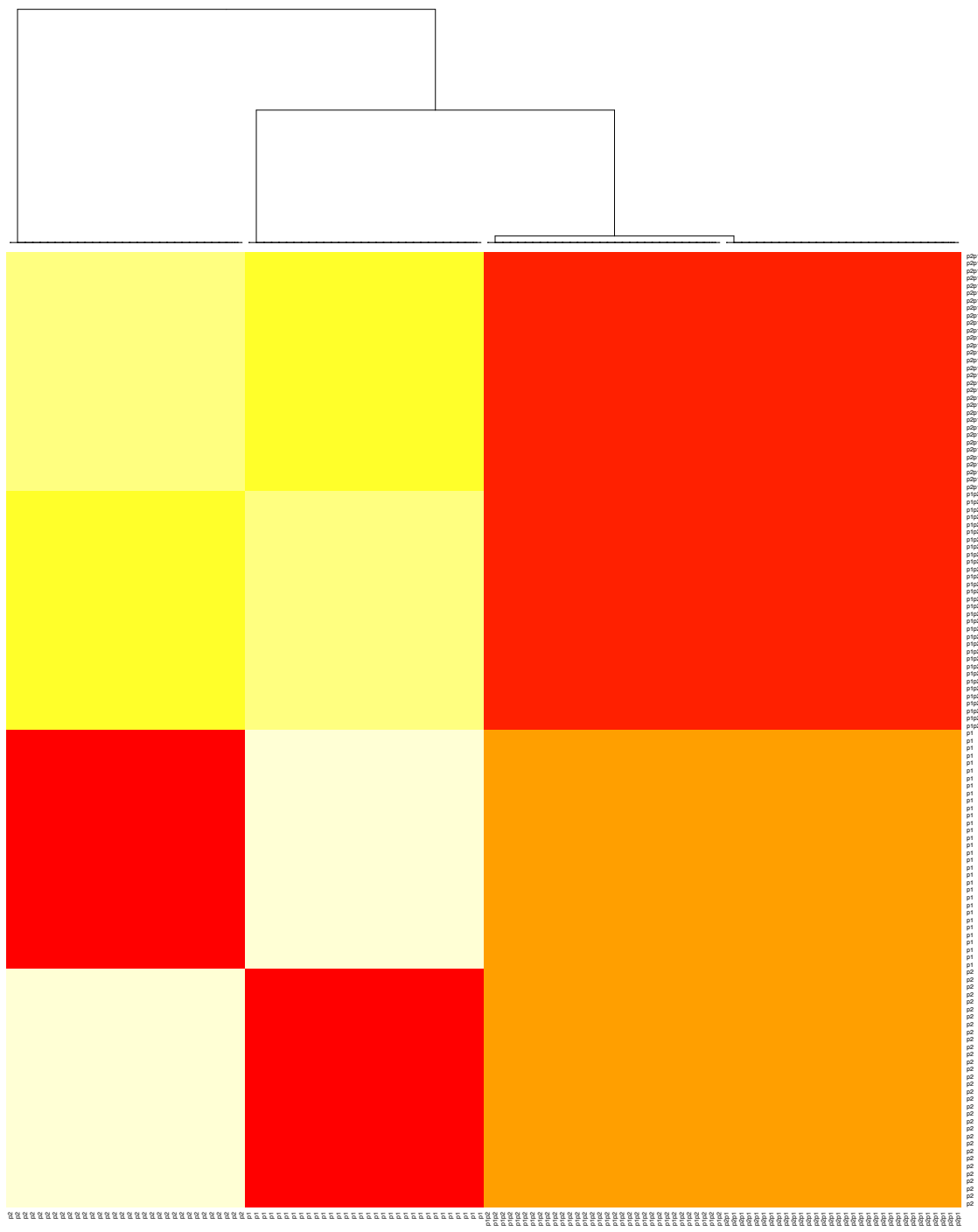

Supplement: Supplementary Figure 2 — Heat plots of A pedigree relatedness matrices from two OP half-sib families (IMC67 and PA121), and two full-sib rootstock families derived from controlled crosses between IMC67 × PA121 and its reciprocal cross, inputed in a “genetic prediction” additive mixed linear model, according to de los Campos et al. (2009). Heat plots are depicted for verification purposes (A) before grafting, and (B) two and (C) four months after grafting with the ICS95 and CCN51 clonal scions under two Cd treatments. [file Image_2.pdf]
